# KDM6A–SND1 interaction maintains genomic stability by protecting the nascent DNA and contributes to cancer chemoresistance

**DOI:** 10.1093/nar/gkae487

**Published:** 2024-06-08

**Authors:** Jian Wu, Yixin Jiang, Qin Zhang, Xiaobing Mao, Tong Wu, Mengqiu Hao, Su Zhang, Yang Meng, Xiaowen Wan, Lei Qiu, Junhong Han

**Affiliations:** Department of Biotherapy, Cancer Center and State Key Laboratory of Biotherapy, Frontiers Science Center for Disease-related Molecular Network, West China Hospital, Sichuan University, Chengdu 610041, China; Department of Biotherapy, Cancer Center and State Key Laboratory of Biotherapy, Frontiers Science Center for Disease-related Molecular Network, West China Hospital, Sichuan University, Chengdu 610041, China; Department of Biotherapy, Cancer Center and State Key Laboratory of Biotherapy, Frontiers Science Center for Disease-related Molecular Network, West China Hospital, Sichuan University, Chengdu 610041, China; Department of Biotherapy, Cancer Center and State Key Laboratory of Biotherapy, Frontiers Science Center for Disease-related Molecular Network, West China Hospital, Sichuan University, Chengdu 610041, China; Department of Biotherapy, Cancer Center and State Key Laboratory of Biotherapy, Frontiers Science Center for Disease-related Molecular Network, West China Hospital, Sichuan University, Chengdu 610041, China; Department of Biotherapy, Cancer Center and State Key Laboratory of Biotherapy, Frontiers Science Center for Disease-related Molecular Network, West China Hospital, Sichuan University, Chengdu 610041, China; Department of Biotherapy, Cancer Center and State Key Laboratory of Biotherapy, Frontiers Science Center for Disease-related Molecular Network, West China Hospital, Sichuan University, Chengdu 610041, China; Department of Biotherapy, Cancer Center and State Key Laboratory of Biotherapy, Frontiers Science Center for Disease-related Molecular Network, West China Hospital, Sichuan University, Chengdu 610041, China; Department of Biotherapy, Cancer Center and State Key Laboratory of Biotherapy, Frontiers Science Center for Disease-related Molecular Network, West China Hospital, Sichuan University, Chengdu 610041, China; Department of Biotherapy, Cancer Center and State Key Laboratory of Biotherapy, Frontiers Science Center for Disease-related Molecular Network, West China Hospital, Sichuan University, Chengdu 610041, China; Department of Biotherapy, Cancer Center and State Key Laboratory of Biotherapy, Frontiers Science Center for Disease-related Molecular Network, West China Hospital, Sichuan University, Chengdu 610041, China

## Abstract

Genomic instability is one of the hallmarks of cancer. While loss of histone demethylase KDM6A increases the risk of tumorigenesis, its specific role in maintaining genomic stability remains poorly understood. Here, we propose a mechanism in which KDM6A maintains genomic stability independently on its demethylase activity. This occurs through its interaction with SND1, resulting in the establishment of a protective chromatin state that prevents replication fork collapse by recruiting of RPA and Ku70 to nascent DNA strand. Notably, KDM6A–SND1 interaction is up-regulated by KDM6A SUMOylation, while KDM6A^K90A^ mutation almost abolish the interaction. Loss of KDM6A or SND1 leads to increased enrichment of H3K9ac and H4K8ac but attenuates the enrichment of Ku70 and H3K4me3 at nascent DNA strand. This subsequently results in enhanced cellular sensitivity to genotoxins and genomic instability. Consistent with these findings, knockdown of KDM6A and SND1 in esophageal squamous cell carcinoma (ESCC) cells increases genotoxin sensitivity. Intriguingly, KDM6A H101D & P110S, N1156T and D1216N mutations identified in ESCC patients promote genotoxin resistance *via* increased SND1 association. Our finding provides novel insights into the pivotal role of KDM6A–SND1 in genomic stability and chemoresistance, implying that targeting KDM6A and/or its interaction with SND1 may be a promising strategy to overcome the chemoresistance.

## Graphical Abstract

**Graphical Abstract:**
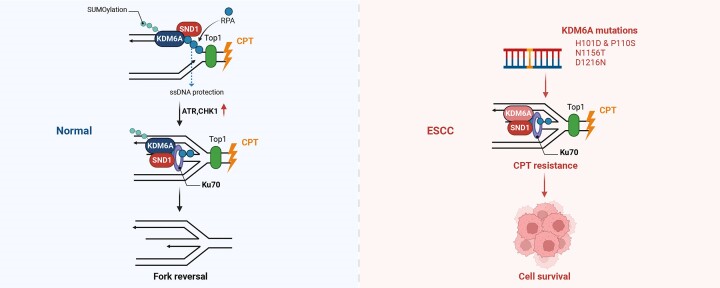

## Introduction

Genomic stability is essential for cell survival and tumorigenesis prevention (1). Throughout the integrated cell cycle, events such as the DNA replication, chromosome separation, euchromatin/heterochromatin conversion, transcriptional activation and nucleosome assembly ensure the dynamic nature of eukaryotic chromatin (2). Eukaryotes have evolved a complicated and rigorous mechanism to maintain genomic stability, ensuring proper cell division and precise DNA replication (3–5). Disruption of genomic stability could trigger DNA damage response (DDR) pathway in cells to maintain the integrity and function of chromatin. This pathway is regulated by multiple protein and signals such as ATM/ATR, BRCA1/2, TP53 (6,7). Emerging evidence reveal the involvement of multiple chromatin remodeling factors in the regulation of genomic stability (8,9). Failure or inhibition of chromatin remodeling leads to DNA damage (10,11). Moreover, chromatin remodeling factors also regulate the DDR directly through histone modification or protein recruiting(12,13). Since the disruption of genomic stability is an important strategy for tumor therapy, it is critical to explore the underlying mechanism.

Replication stress is one of main endogenous factors that induces genomic instability. In response to replication stress, the leading and lagging DNA chains quickly flipped under the mediation of Rad51 and RPA protein, resulting in the formation of DNA double strands with sticky ends. This structure could protect the function of replication forks (14,15), which is also known as Holliday junction and instrumental in forks stability. Similar to regular the ordinary DNA double strands, nucleosome assembly could also occur in the reversed nascent DNA, effectively preventing DNA degradation by nuclease (16,17). During this process, histone modification and chromatin remodeling factors play a critical role in protecting nascent DNA. For instance, histone demethylase KDM5B could recognize double-strand break DNA and initiate DNA repair through recruiting Ku70 and BRCA1 proteins to DNA breaks (18). Histone methylase SETD1A could protect newly synthesized DNA from degradation by exonuclease *via* catalyzing histone methylation of nascent double-strand DNA (19). However, the involvement of other histone modifications and chromatin remodeling factors in the remodeling of replication forks remains largely elusive.

Histone demethylase 6A (KDM6A), located on the X chromosome, is a member of the chromatin remodeling factors(20). Jumonji C (JmjC) domain in the C-terminal of KDM6A specifically catalyzes the demethylation of lysine at position 27 of histone H3. Moreover, a tetratricopeptide repeat (TPR) domain in N-terminal of KDM6A mainly mediates the protein-protein interaction (PPI) (21,22). Furthermore, additional evidence reveals that KDM6A is closely related to embryonic development and tumorigenesis (23). For instance, high tumor incidence is observed in KDM6A knockout mouse (24). Whereas KDM6A mutation has been identified in multiple tumors, promoting tumorigenesis and progression, such as in bladder cancer, renal cell carcinoma (25,26). Evidence indicates that the loss of function of KDM6A could increase the H3K27me3 level at the promoter region of multiple tumor suppressor genes, subsequently promoting tumor malignancy. The catalytic inactivation induced by mutation in JmjC or TPR domain can also abolish the tumor suppressive function of KDM6A, suggesting that KDM6A suppresses tumorigenesis independent of its histone demethylase capability (27,28). Genomic instability is a classical hallmark of cancer. Disruption of genomic stability could lead to chromosome breakage, translocation, and gene mutation, which are critical for tumorigenesis. As an important chromatin remodeling factor, KDM6A deficiency has been proved to increase the risk of tumorigenesis. Thus, we speculate that KDM6A play a pivotal role in maintaining genomic stability and preventing tumorigenesis and tumor progression. Actually, convincing evidence have demonstrated that KDM6A not only catalyzes H3K27 demethylation directly by JmjC domain, but also fulfill its function through PPI and phase separation mediated by TPR domain or intrinsic disorder region (IDR) respectively (29,30), indicating that KDM6A might regulate the genomic stability in a demethylase dependent and/or independent manner (31). Thus far, the involvement of KDM6A in the regulation of replication fork stability remains largely unknown.

In current study, we show that KDM6A interacts with SND1, a chromatin architectural modifier. The interaction between KDM6A and SND1 is enhanced by KDM6A SUMOylation, while KDM6A K90A mutation almost abolishes this interaction. In addition, KDM6A knockdown impairs genomic stability by suppressing RAP32 phosphorylation and reducing γH2AX expression. This disruption also affects its interaction with SND1 and reduces the enrichment of Ku70 and RPA1 enrichment at nascent DNA, eventually resulting in DNA damage. Moreover, KDM6A H101D & P110S, N1156T and D1216N mutations identified in esophageal squamous cell carcinoma (ESCC) patients showed the increased interaction with SND1 and enhanced resistance to genotoxin. Conversely, knockdown of both KDM6A and SND1 elevates the sensitivity to genotoxin. Collectively, our study shed light on the novel mechanism by which KDM6A influences genomic stability and drug resistance in ESCC.

## Materials and methods

### Plasmid construction

Two specific shRNA sequence targeting KDM6A were synthesized by You Kang biotechnology company. For plasmid construction, the shRNAs were cloned into pLKO.1.TRC vector predigested by AgeI and EcoRI. HA-KDM6A, HA-KDM6A H1146A, and the truncation mutants including HA-KDM6A1-400aa, HA-KDM6A 400–1000aa and HA-KDM6A 1000–1401aa plasmids were constructed using a standard approach. Wild-type and truncation mutant SND1 plasmids were constructed into pLv-3Myc and pLv-GFP vector using cDNA amplified from HEK293T cells respectively. RPA1 and SUMO1 plasmids were also constructed into pLv-3Flag and pLv-GFP vector using cDNA from HEK293T cells. The sequence of shRNA and primers were listed in Table 1.

**Table 1.** shRNAs and primers used in the study

**Primer**	5′ to 3′
*sh-KDM6A-1-F*	CCGG GCAGCACGAATTAAGTATTTA CTCGAG TAAATACTTAATTCGTGCTGC TTTTTG
*sh-KDM6A-1-R*	AATTCAAAAA GCAGCACGAATTAAGTATTTA CTCGAG TAAATACTTAATTCGTGCTGC
*sh-KDM6A-2-F*	CCGG GATGCAAGTCTATGACCAATT CTCGAG AATTGGTCATAGACTTGCATC TTTTTG
*sh-KDM6A-2-R*	AATTCAAAAA GATGCAAGTCTATGACCAATT CTCGAG AATTGGTCATAGACTTGCATC
*sh-SND1-1-F*	CCGG GCTGATGATGCAGACGAATTT CTCGAG AAATTCGTCTGCATCATCAGC TTTTTG
*sh-SND1-1-R*	AATTCAAAAA GCTGATGATGCAGACGAATTT CTCGAG AAATTCGTCTGCATCATCAGC
*sh-SND1-2-F*	CCGG GAAGGCATGAGAGCTAATAAT CTCGAG ATTATTAGCTCTCATGCCTTC TTTTTG
*sh-SND1-2-R*	AATTCAAAAA GAAGGCATGAGAGCTAATAAT CTCGAG ATTATTAGCTCTCATGCCTTC
KDM6A H1146A-F	AGGTgcTCAGGAAAATAACAACTTCTGTTCAGTTAACATAAATAT
KDM6A H1146A-R	CCTGAgcACCTGGTGTTCTGCTCCCTGGAACT
KDM6A^H101D & P110S^-F	GGTGACTTCAACCTCTTATTGGAAGATTATTCAAAAGCATTA
KDM6A^H101D & P110S^-R	TAATGCTTTTGAATAATCTTCCAATAAGAGGTTGAAGTCACC
KDM6A ^L259M^-F	CTGTAGATCTCATGGGAGATAAAGCCACCA
KDM6A ^L259M^-R	TCTCCCATGAGATCTACAGTGTGATGCATCCA
KDM6A ^N1156T^-F	AGGAAACTAACAACTTCTGTTCAGTTAACATAAATATT
KDM6A ^N1156T^-R	TGTTAGTTTCCTGATGACCTGGTGTTCTGCTCC
KDM6A ^D1216N^-F	CCTGGAAATTTGGTCTGGATAAATGC
KDM6A ^D1216N^-R	CCAAATTTCCAGGTCGCTGAATAAACCTA
KDM6A ^P1243dup^-F	TTGGTCCACCACTTACAGCCTGCCAGTATAAATTG
KDM6A ^P1243dup^-R	GGCTGTAAGTGGTGGACCAACATTCCAAGCAATGT
3Myc-SND1-F	CTGTGGCAGAGGTAAAACTTC
3Myc-SND1-R	GACAAGGTAACCCAGGTAGAAG
Flag-RPA1-F	TGACAAGCTCGAGATGGTCGGCCAACTGAGCGAG
Flag-RPA1-R	AACTAGTCTCGAGTCACATCAATGCACTTCTCCTG
KDM6A 1-400aa-F	GTTGTAGTTGAAAGGGCGAATTCGCGGCCGCACTCG
KDM6A 1-400aa-R	CCTTTCAACTACAACTTTTGCTTCTAGTTGCATTTAAG
KDM6A^400-1000 aa^-F	CTTCTGCAGGAATTCTGTAAACCTCATCATCCAAATAC
KDM6A^400-1000 aa^-R	CTGTCGACGATATCTTAGTTAGCTTCCACCAAAGTTTTAG
KDM6A^1000-1401 aa^-F	TACGCTCTCGAGCCCATGGATGCTTTACAGGCCTATA
KDM6A^1000-1401 aa^-R	ACTAGTCTCGAGTCAAGATGAGGCGGATGGTAATGGA
Myc-SND1 SN del-F	AGGGTGCTATGAGGAGCAGCCCGTGGAGGAGGTGAT
Myc-SND1 SN del-R	TCCTCATAGCACCCTGAGAGGACCATCTTGATGATGC
Myc-SND1 TSN del-F	CTCCTATTAACTCGAGACTAGTTCTAGAGCGGCC
Myc-SND1 TSN del-R	TCGAGTTAATAGGAGCCCTCTACAGGGGGGTGACT
GFP-SND1-F	AGGGTCCCTCGAGAATGGCGTCCTCCGCGCAGAG
GFP-SND1-R	GAATTAGTCTCGAGTTAGCGGCTGTAGCCAAATTCG
GFP-SND1 SN del-F	GGTCCCCTATGAGGAGCAGCCCGTGGAGGAGGT
GFP-SND1 SN del-R	TCCTCATAGGGGACCGCGGGTCCCCCGGAGGA
GFP-SND1 TSN del-F	GGGCTCCTATTAACTCGAGACTAATTCTAGAGC
GFP-SND1 TSN del-R	AGTTAATAGGAGCCCTCTACAGGGGGGTGACTG
GFP-RPA1-F	TTCCGGTGAATTCATGGTCGGCCAACTGAGCGAG
GFP-RPA1-R	ACACCATGAATTCCATCAATGCACTTCTCCTGATGCTC

### Cell viability assay and colony formation assay

The indicated cells were inoculated into 24-well plates at a density of 2 × 10^5^ and cultured overnight in cell incubator with 5% CO_2_ and 37ºC atmosphere. Once the cells completely adhered to the plate, 1 μM of CPT was added into culture for 4 h. The cells were rinsed twice with precooling PBS and surviving cells was determined by crystal violet staining 24 h later. Colony formation assay was conducted according to previously published protocol (32). Briefly, 1 × 10^3^ cells were seeded in 6-well plates and co-cultured with 1 μM of CPT for 4 h. After replaced the fresh medium, the cells were cultured for 10 days and detected by crystal violet staining.

### Chromosomal breakage analysis

Cells were inoculated at 3 × 10^5^ per well into 6-well plates and treated with 1 μM of CPT for 24 h; And then indicated cells were exposed to 1 μg/ml colchicine for 4 h and treated with 75 mM of KCl solution for 20 min, 3:1 methanol/acetic acid was added to fix cells. Collected the cell suspension was dropped on the precooled slide with a straw (soak in 80% ethanol and store in 4ºC refrigerator). Slides were stained with DAPI, and images were captured by laser confocal microscope. Thirty metaphase spreads were randomly chosen for aberrations analysis for each group.

### Cell cycle profiling

Cells cycle was analyzed by the PI/RNase Staining buffer kit (BD company, USA) and operated strictly in accordance with the instructions. Briefly, 1 × 10^6^ of HeLa or HEK293T cells were collected and rinsed twice with PBS buffer. Precooled 70% ethanol was added to fix the cells overnight at 4°C. After washing the cells pellets with PBS, PI/RNase staining buffer was added for about 20 min. Cell cycle was profiled by flow cytometry cell sorting.

### Apoptosis analysis

5 × 10^5^ indicated cells were inoculated into 6-well plates and treated with 1 μM of CPT for 12 h. Cell pellets was collected by centrifuge and resuspend into 100 μL PBS buffer, after added 5 μL of PE Annexin V and 5 μL of PI buffer, cells were incubated at room temperature for 15 min without light. After added 300 μL of PBS buffer to each sample, the sample was subjected to flow cytometry cell sorting.

### Co-immunoprecipitation and mass spectrometry

Cell pellets were collected and washed twice with cold PBS buffer followed by mixed with lysis buffer (20 mM HEPES (pH 8.0); 150 mM NaCl; 1 mM EDTA; 1.5 mM MgCl_2_; 1% Triton X-100; 10% glycerol) supplemented with protease inhibitor cocktail (Roche) and 1% PMSF. The lysate was placed in a non-contact ultrasonic instrument for crushing with conditions: 30% power, 5 s ultrasonic, 5 s interval, repeat 3–5 times. After centrifuge at 12000 g, 4 ºC and added magnetic beads into the lysate, and the samples was incubated in a shaker at 4ºC for 2 h. The magnetic beads were recovered by sitting on magnetic rack and were washed 3 times with washing buffer (20 mM HEPES, pH 8.0); 150 mM NaCl; 1 mM EDTA; 0.5% Triton X-100; 10% glycerol, 1% PMSF). After mixed the beads with 1 × loading buffer and boiled for 10 min, samples were subjected to SDS-PAGE electrophoresis and silver staining. The coimmunoprecipitated proteins were identified by mass spectrometry (PTM biolabs, China).

### Nuclear and cytoplasmic protein separation

The separation of nuclear and cytoplasmic protein was performed according to the protocol published previously. In brief, about 3 × 10^6^ cells were collected and washed with pre-cooled PBS twice. After that, the cell pallet was dissolved in 200 μL of buffer A (10 mM HEPES, 10 mM KCl, 1.5 mM MgCl_2_, 0.34 M Sucrose, 10% glycerol, 1 mM dithiothreitol, 0.1% Triton X-100, pH 7.9) with 1% PMSF on ice for 5 min. Nuclear protein (P1) were obtained by 1500 g centrifugation for 4 min at 4ºC; After washing the nucleus once with buffer A without 0.1% Triton X-100, the sample was dissolved in buffer B (3 mM EDTA, 0.2 mM EGTA, 1 mM dithiothreitol and 1% PMSF). After incubation on ice for 10 min, soluble nucleoprotein (S1) was isolated from chromatin by centrifugation at 2000 g for 4 min; The separated chromatin binding protein (P2) was washed once with buffer B and centrifuged at 13000 g for 1 min. After that, P2 was mixed with 1 × loading buffer and boiled for 10 min. The chromatin binding protein was detected by western blot.

### iPOND assay

iPOND was performed in HEK293T and Kyse150 cells respectively as previously described (33,34). Briefly, indicated cells were seeded in 10 cm dish. After cell density reached 80%, 10 mM thymidine was added into cell culture and incubated for overnight to arrest cell at G1/S boundary, prior to DNA replication. 10 μM of EdU was added and incubated for 30 min; After treatment with 1 μM of hydroxyurea for 4 h, cells were fixed at room temperature for 20 min with 10 mL of 1% formaldehyde; The crosslink was stopped by 1.25 M glycine. After added the Click-reaction cocktails (1 × PBS, 10 μM Biotin-azide, 10 mM Sodium ascorbate, 2 mM CuSO_4_) for coupling the biotin to EdU while rotating reactions at RT for 1–2 h, collected cell pellets were resuspended with lysis buffer. The resulted sample was subjected to ultrasonication under the conditions: 13–16 W, 20 s ultrasonic, 40 s interval, repeat ultrasonication cycle for 5 times. After centrifugation at 14000 g for 10 min at 4ºC, the collected supernatant was incubated with streptavidin magnetic beads to capture the DNA-EdU complex while shaking at 4ºC overnight. After washing for 3 times with washing buffer, the beads were mixed with 1 × loading buffer and boiled for 10 min. The samples were subjected to western blot, and the proteins was detected using specific antibodies against the protein indicated in figures.

### DNA fiber spreading assay

DNA fiber spreading assay was performed using the method described earlier. In brief, indicated cells were co-cultured with 20 μM of 5-chloro-2-deoxyuridine (CldU) for 20 min with the condition of 37 °C and 5% CO_2_, washed three times with pre-warmed 1 × PBS, and then treated with 1 mM of HU for 4 h to induce the replication stress. After washing three times with pre-warmed 1 × PBS, indicated cells were harvested by trypsinization and resuspended in pre-cool PBS (2 × 10^5^ cells/ml). Then, 2 μL of cell suspension was mixed with 8 μL of lysis buffer [200 mM Tris–HCl (pH 7.5), 50 mM EDTA, 0.5% (w/v) SDS] on a glass slide by gently stirring with a pipette tip. After 10 min incubation at room temperature, the slides were tilted at 150, the drops was glide down the slides slowly under the gravity. After leaving the DNA spreads air-dried for 30 min at least, DNA fiber were fixed in methanol/acetic acid (3:1) at room temperature for 10 min and air-dried for 30 min. After that, DNA spreads were denatured with 2.5 M HCl for 1 h at RT followed by rinsing 3 times with 1 × PBS and blocked with 1 × PBS plus 2% BSA and 0.5% tween 20 for 1 h at room temperature. Dilute rat monoclonal anti-BrdU in the ratio of 1:500 (anti-CldU ab6326) and apply 100 μL of diluted primary antibody to each slide, and then incubate 2 h at room temperature followed by rinsing 3 times with 1 × PBS. Dilute Goat anti-rat IgG Alexa Fluor 488 in the ratio of 1:500 in blocking buffer and apply 100 μL per slide which was incubated for 1 h in the dark. Rinse 3 times in PBS and air dried overnight in dark. Images were acquired with a Leica p38 fluorescent microscope. CldU tract lengths (μm) were measured using ImageJ.

### Immunofluorescence staining assay

HeLa cells were seeded on cell climbing sheet at the density of 1 × 10^5^. Cells were fixed at room temperature for 10 min with 4% paraformaldehyde, followed by permeabilization with 0.5% Triton X-100 for 5 min and blocking with 10% goat serum for 30 min at room temperature. After rinsed for 3 times with TBST buffer, the staining was performed with the primary antibody overnight at 4°C while fluorescent antibody staining was performed at room temperature for 1 h. For phospho-RPA^S4/8^ and γH2AX foci detection, cells with knockdown of KDM6A and SND1 were treated with 1 μM of CPT for 2 h before fixation. For protein co-localization observation, cells were transfected with indicated plasmid before treatments. For observing the co-localization signal between KDM6A/SND1 and nascent DNA, indicated cells were co-cultured with 10 μM of EdU for 10 min followed by HU treatment. For investigate the BrdU and EdU foci formation, indicated cells were co-cultured with 1 mM BrdU for 10 min and then with 10 μM of EdU for 10 min followed by HU treatment. To evaluate the restart of replication forks, indicated cells were co-cultured with 20 μM of BrdU for 10 min followed by HU treatment and then co-cultured with 10 μM of EdU for 10 min. At least 30 cells were randomly selected to count the number of phospho-RPA^S4/8^, γH2AX, BrdU and EdU foci. Images were captured using a Zeiss AX10 fluorescence microscope and AxioVision software.

### Antibodies

The antibodies used in this study include anti-KDM6A (for IB: CST, 1:1000; for IF:1:200); anti-SND1 (Cell Signaling, 1:1000), anti-H3K27me3 (CST, No. 9733, 1:2000), anti-H3K9ac (CST, No. 9733, 1:2000), anti-H4K8ac(CST, No. 2594, 1:1000), H3 (CST, clone FL136,1:2000), anti-Ku70 (Santa Cruz, clone B7, 1:1000), anti-PCNA (clone PC-10, 1:1000), anti-FLAG (CST, 1:2000), anti-HA (CST, 1:2000), anti-Myc (CST, 1:2000), anti-RPA pSer4/8 (CST, 1:1000 and 1:200), anti-RPA pSer33 (Novus, 1:1000), anti-γH2AX (CST, 1:1000 and 1:200). Secondary antibodies used for immunoblotting were HRP-linked anti-mouse or rabbit (Life Technologies, 1:5000) while for immunofluorescence we used AlexaFluor antibodies (Life Technologies, 1:1000).

### Xenograft model

All animal procedures were approved by the Sichuan University Institutional Animal Care and Use Committee protocols. 16 female BALB/c‐nu mice (5 weeks of age, 18–22 g) were purchased from Tengxing company (Chongqing, China) and kept in barrier facilities for a 12 h light/dark cycle. All experimental procedures were approved by the Institutional Animal Care and Use Committee of Sichuan University. Kyse150 cells were divided into 4 groups: sh-scramble, shKDM6A, KDM6A^mut^ and KDM6A^wt^, and subcutaneously injected into the left armpit of male nude mice at the density of 5 × 10^6^ per group. Once small nodules were observed at the inoculation site of nude mice, 1 mg/kg CPT was injected intraperitoneally, and given every 4 days thereafter. Tumor size was measured with calipers and calculated with the formula: volume (mm^3^) = [width^2^ (mm^2^) × length (mm)]/2. At day 28, tumors were dissected and weighed.

### Cell culture and transfections

HeLa and HEK 293T were preserved by our lab and maintained in DMEM plus 10% fetal calf serum (FCS) with penicillin–streptomycin (1%). Before transfection, cells were seeded in 6-well plate at a density of 5 × 10^5^, 1 mL of virus harboring sh-Scramble or sh-KDM6A or sh-SND1 were added into indicated cells for 24 h, and stable interference cell lines were screened by 2 μg/mL of puromycin for 7 days. For gene expression, 2 μg of HA-KDM6A, HA-KDM6A^H1146A^, HA-KDM6A^1-400aa^, HA-KDM6A^400-1000 aa^, HA-KDM6A^1000-1401 aa^, pLv-3Myc-SND1, pLv-3Myc-SNΔ, pLv-3Myc-TSNΔ, pLv-3FLAG-RPA1, pLv-GFP-RPA1 were transfected into HEK293T and HeLa via PEI respectively. For SUMOylation detection, 2 μg of pLv-3FLAG-SUMO1 were transfected into HEK293T cells together with 2 μg of HA-KDM6A or pLv-3Myc-SND1 using PEI.

## Results

### Histone demethylase KDM6A is critical for genomic stability

**Figure 1.**
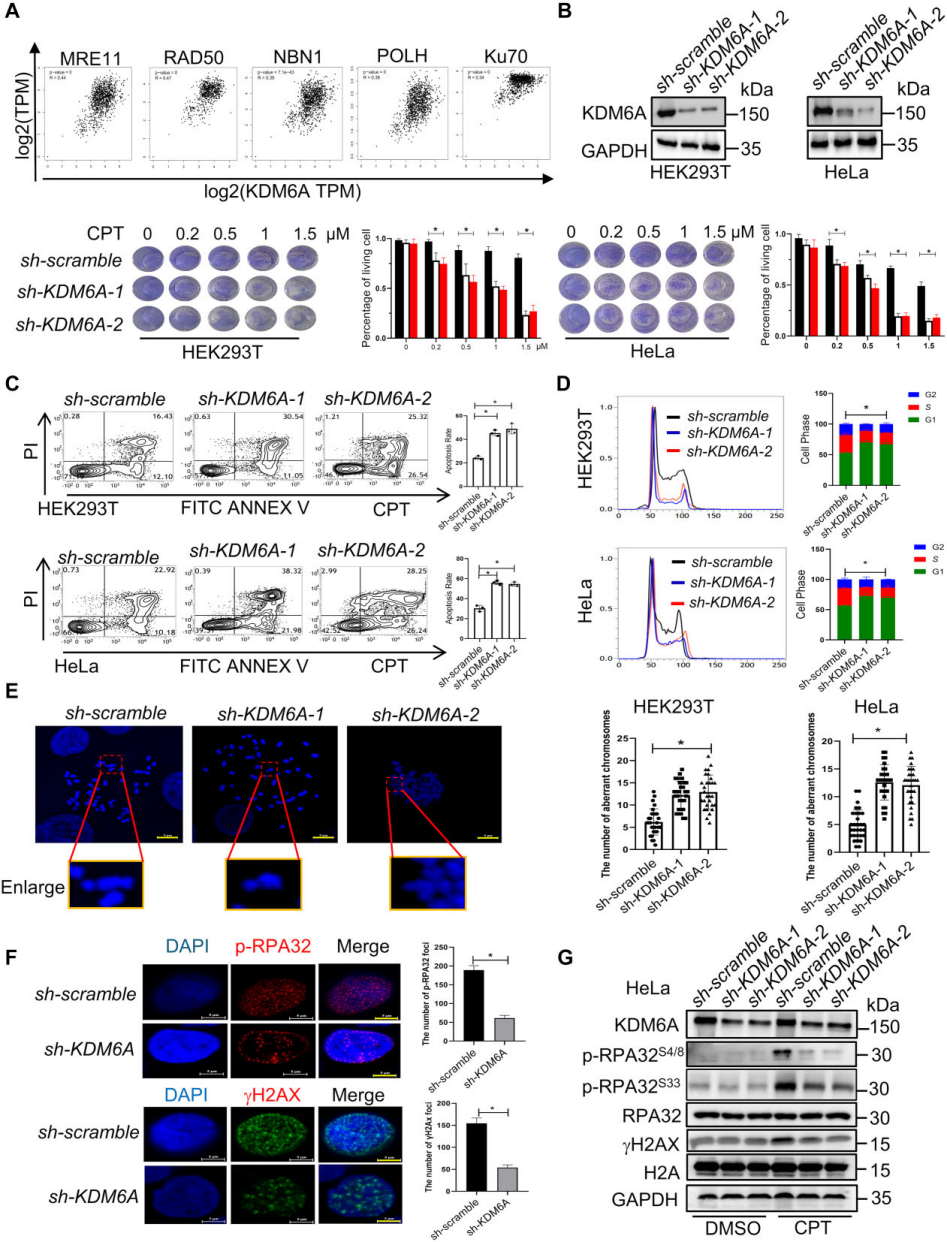
Loss of KDM6A triggers the genomic instability in HEK293T and HeLa cells. (**A**) The correlation between KDM6A and DNA damage-related genes is analyzed using the dataset from GTEx database. (**B**) Western blot and cellular viability analysis in HEK293T and HeLa cells transfected with scramble or KDM6A shRNA plasmids. (**C**) Apoptosis for indicated cells with KDM6A knockdown in the presence of genotoxin CPT. (**D**) Cell cycle profile for indicated cells with KDM6A knockdown. (**E**) Abnormal chromosome in indicated cells with KDM6A knockdown. (**F**) Image of phospho-RPA32 (S4/S8) and γH2AX foci was captured by fluorescence microscopy. Representative images with scale bars representing 5 μm and quantifications are shown. (**G**) The expression of phospho-RPA32 (S4/S8 and S33) and γH2AX in HeLa cells was detected by western blot in indicated cells. The value of histogram in (B-D) and (G) represents mean data ± S.E. from three independent experiments, *P < 0.05. Significant *P* values are indicated and were determined using a paired Student's *t* test.

Since both the enhanced transcriptional activity and histone methylation influence the genomic stability, we hypothesize that KDM6A is involved in maintaining genomic stability. To test this hypothesis, we first analyzed the correlation between KDM6A expression and DNA damage pathway in normal tissues using GTEx database. Indeed, KDM6A expression was positively correlated with multiple DNA repair related proteins such as MRE11, RAD50, NBN1, POLH and Ku70 (Figure 1A), indicating a potential correlation between KDM6A and DNA damage. To assesses determine the influence of KDM6A on genomic stability, we knocked KDM6A down in cells and observed obvious cell death in the presence of camptothecine (CPT), an inhibitor of DNA topoisomerase I (Figure 1B), along with increased apoptosis (Figure 1C), suggesting that loss of KDM6A enhances the sensitivity to CPT. In addition, KDM6A knockdown also led to G1/S phase arrest in HEK293T and HeLa cells, indicating that KDM6A might be involved in regulating DNA replication (Figure 1D). Since CPT also cause double strand DNA damage during the S phase, we investigated the abnormal chromosome in cells exposed to CPT by quantifying the number of chromosomes with breakage or gaps. The results showed that the number of aberrant chromosomes in cells lacking KDM6A was significantly higher than that in the control, suggesting that loss of KDM6A indeed impairs genomic stability (Figure 1E). Genomic instability, particularly under DNA damage and/or replication stress, often leads to the activation of ATM and ATR signaling pathway. During DNA repair and DNA replication, RPA complex, consisting of RPA1, RPA2/RPA32 and RPA3, is recruited to coat single strand DNA to prevent it from degradation (35–37). Therefore, we further detected the phosphorylated-RPA32^S4/S8^ and γH2AX expression in addition to foci formation by western blot and immunofluorescence staining respectively. The results showed that the foci formation and the expression of phosphorylated-RPA32^S4/S8^ and γH2AX were both obvious reduction in cells with KDM6A knockdown compared to that in the control after CPT treatment (Figure 1F, G), implying that KDM6A may play a critical role in DNA damage response (DDR), even though the expression of phosphorylated-RPA32^S33^ also showed slight reduction. Together, these data demonstrate that loss of KDM6A enhances the sensitivity to CPT and impairs DDR, suggesting a substantial contribution of KDM6A to maintain genomic stability.

### KDM6A regulates genomic stability mainly in a demethylase-independent manner.

**Figure 2.**
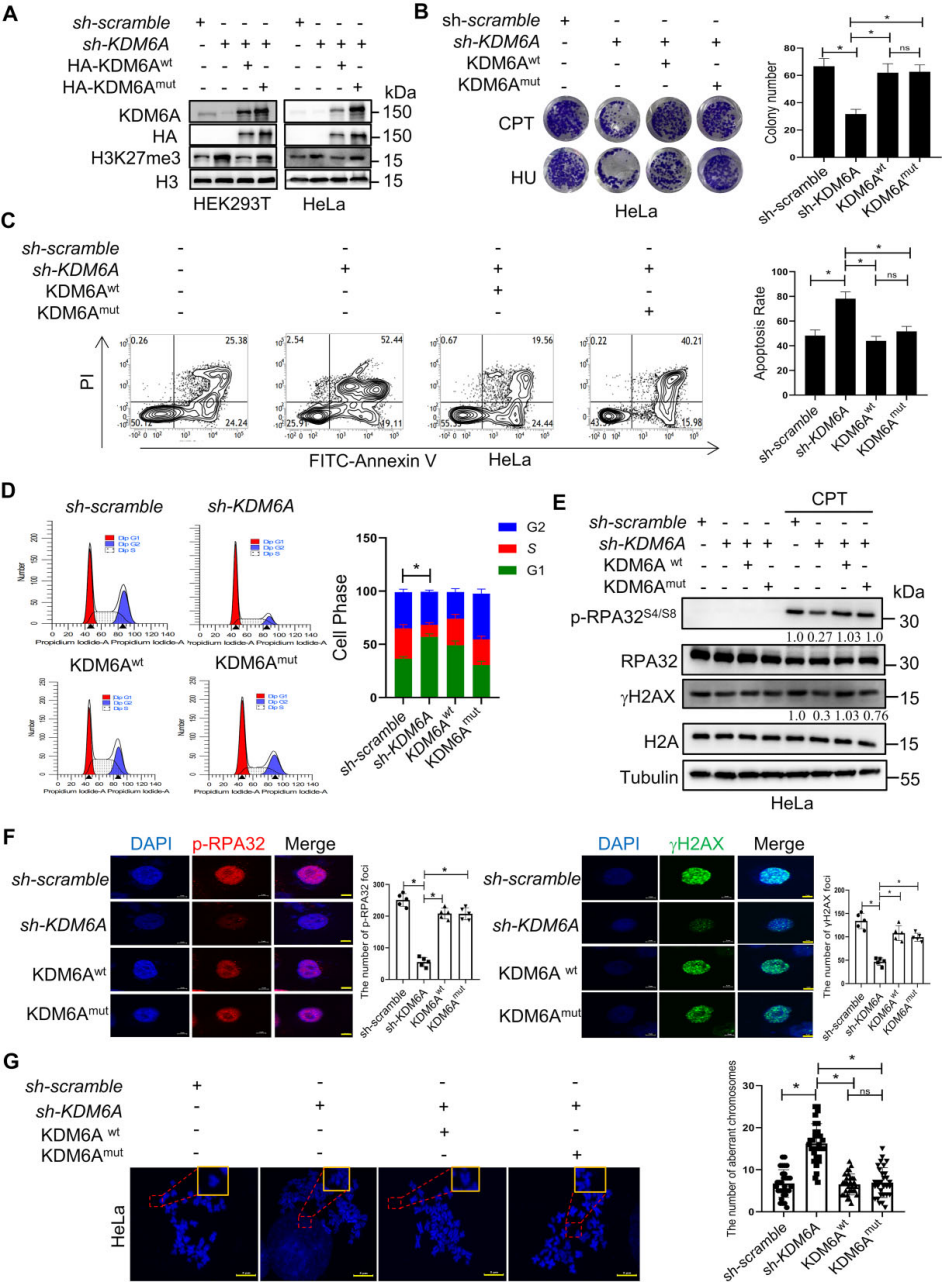
KDM6A regulates the genomic stability independently on its demethylation activity. (**A**) Western blot with the indicated antibodies in HEK293T and HeLa cells knocked down endogenous KDM6A and followed by transfection with the plasmid of KDM6Awt or catalytic-dead KDM6Amut respectively. (**B**) Cellular viability for genotoxin CPT or HU treated HeLa cells which is used in panel A. (**C**) Apoptosis determined by flow cytometry for HeLa cells used in panel A in the presence of CPT. (**D**) Cell cycle profile for HeLa cells used in panel A. (**E**) Western blot for phospho-RPA32 (S4/S8) and γH2AX in HeLa cells used in panel A. Phospho-RPA32 (S4/S8) and γH2AX expression were normalized to RPA32 and γH2AX expression respectively and the quantification number was labeled below the protein bands. (**F**) Image of phospho-RPA32 (S4/S8) and γH2AX foci was captured in HeLa cells used in panel A by fluorescence microscopy. Representative images with scale bars representing 5 μm and quantifications are shown. (**G**) Abnormal chromosome for the indicated HeLa cells. Representative images and quantifications are shown. Scale bars, 5 μm. All experiments were independently repeated three times, *P < 0.05. *P* values were determined using a paired Student's *t* test.

Convincing evidence have revealed that KDM6A exerts its biological function in a manner dependent on or independent of its histone demethylase. To determine whether KDM6A regulated genomic stability depends on its histone demethylation activity, the recombinant KDM6A expression plasmid was transfected into HEK293T and/or HeLa cells lacking endogenous KDM6A expression. The results showed that HA-tagged wild-type KDM6A almost abolished the increased H3K27me3 expression in endogenous KDM6A-deficient cells while the catalytic-dead HA-KDM6A (H1146A) failed to do so, indicating that recombinant KDM6A variants function well in cells (Figure 2A). We also found that exogenous HA-KDM6A and KDM6A H1146A protein could remarkably rescue the survival of cells with KDM6A knockdown under exposure to CPT or HU (Figure 2B). Besides, apoptosis levels were comparable between cells expressing wild-type and catalytic-dead KDM6A, but significantly lower than those in cells lacking endogenous KDM6A (Figure 2C). In addition, both exogenous HA-KDM6A and KDM6A H1146A protein rescued the cell cycle from the G1/S arrest caused by KDM6A knockdown (Figure 2D). To further investigate whether the activity of KDM6A is required for the activation of ATR and ATM pathway, we detected the expression of phosphorylated RPA32^S4/S8^, γH2AX, and the foci formation. The results showed that both HA-KDM6A or KDM6A H1146A not only restored the expression of RPA32^S4/S8^ phosphorylation and γH2AX from the suppression, but also the foci number of phospho-RPA32^S4/S8^ and γH2AX in cells with KDM6A knockdown, suggesting that the regulation on DDR is independent on KDM6A demethylase capability (Figure 2E and F). Furthermore, we evaluated the aberrant chromosomes in cells treated with CPT and found that the number of breakage and gap on chromosomes in shKDM6A cells with the expression of HA-KDM6A or KDM6A H1146A was much lower than those in sh-KDM6A cells, and there was not obvious difference between HA-KDM6A or KDM6A H1146A group, suggesting that the involvement of KDM6A in genomic stability maintenance and KDM6A enzyme activity probably has a minor role in the regulation on genomic stability (Figure 2G). Taken together, these data imply that KDM6A possibly regulates the genomic stability in a demethylase-independent manner.

### KDM6A maintains the genomic stability through protecting DNA replication forks against collapse

**Figure 3.**
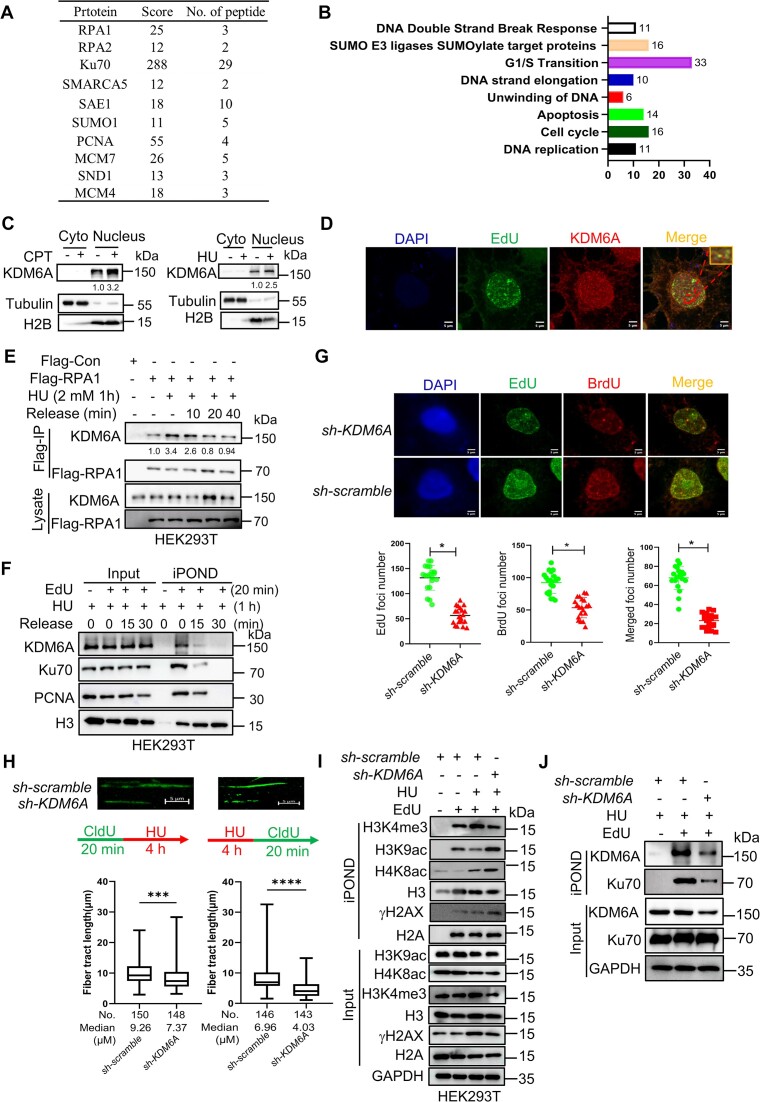
KDM6A influence the genomic stability through regulating the stability and restart of DNA replication forks. (**A**) Copurified SND1 and DNA replication related proteins with KDM6A were validated by mass spectrometry and listed in table. (**B**) Interaction network of replication forks related proteins coimmunoprecipitated with KDM6A. (**C**) Detection of the chromatin associated KDM6A. Chromatin-associated proteins were prepared from the nucleus of HEK293T cells treated with either CPT or HU and were subjected to western blot. The number represents the quantification of KDM6A against to H2B. (**D**) Detection of KDM6A associated with nascent DNA in HeLa cells treated with CPT. Representative images are shown. Scale bars, 5 μm. (**E**) Western blot of RPA1 immunoprecipitation in HEK293T cells treated with HU for 1 h and followed by releasing into fresh media for indicated time. Copurified proteins were detected using specific antibodies. The number represents the quantification of KDM6A protein against to Flag-RPA1. (**F**) Western blot of nascent DNA-associated proteins at stalled replication forks. Nascent DNA-associated proteins were isolated in HEK293T cells treated with HU for 1 hour and followed by releasing into fresh media for indicated time using iPOND approach, and the resulted proteins were detected using specific indicated antibodies. (**G**) The replication forks foci labeled by EdU and BrdU in HeLa cells with HU. Representative images are shown. The histogram counted the number of EdU and BrdU foci in KDM6A-knockdown HeLa cells. Scale bars, 5 μm. * *P* < 0.05. *P* values were determined using a paired Student's *t* test. (**H**) The progression (left panel) and restart (Right panel) of replication forks were evaluated by DNA fiber assay. The histogram counted the length of DNA fiber labeled by CldU in KDM6A-deficient HeLa cells. (I, J) Detection of H3K9ac, H4K8ac, γH2AX (**I**) and Ku70 (**J**) associated with the nascent DNA in cells after KDM6A knockdown.

It has been proven that KDM6A could regulate histone modification and transcription through PPI. For instance, KDM6A regulates the enhancer activity *via* H3K4me and H3K27ac as a component of COMPASS complex. There are increasing evidence showing that KDM6A could influence the transcription by recruiting transcription factors. Here, we hypothesize that KDM6A may also regulates genomic stability through PPI as well. To verify our hypothesis and explore the underlying mechanisms, we performed KDM6A co-immunoprecipitation (Co-IP) followed by mass spectrometry to identify the captured proteins. Total 591 proteins captured by KDM6A were identified through mass spectrometry. Among these, we found that 11 of 591 proteins were involved in DNA replication, including RPA1, RPA2, RPA3 and PCNA; 33 proteins, including members from the MCM family, were associated with cell cycle regulation; 16 proteins were related to protein SUMOylation, including SUMO1 and SAE1. In addition, multiple DDR/DNA replication-associated proteins including Ku70/Ku80, SMARCA5 and SSRP were also identified (Figure 3A and B). These results indicated that KDM6A probably influenced DNA replication. To determine the role of KDM6A in DNA replication, we first induced replication forks stalling using CPT and HU respectively. We observed a significant increase in chromatin-associated KDM6A after CPT or HU treatment (Figure 3C). To detect whether KDM6A could be recruited to nascent DNA under replication stress, we investigated the co-localization between nascent DNA labeled with EdU and KDM6A. Results showed that KDM6A colocalizes with nascent DNA, suggesting that KDM6A is probably involved in the regulation of DNA replication (Figure 3D). As we known, RPA1 could prevent nascent DNA strand from degradation by nuclease through coating it to single-strand DNA. To determine whether KDM6A were involved in maintaining the stability of replication forks through RPA1, we investigated the interaction between RPA1 and KDM6A using co-IP and immunofluorescence staining assay respectively. Indeed, we observed the co-localization signal between GFP-RPA1 and KDM6A in the cell nucleus (Supplementary Figure S1). Interestingly, we also found that KDM6A were captured with RPA1 in the indicated cells with Flag-RPA1 expression (Figure 3E). Additionally, the co-immunoprecipitation signal of KDM6A were significantly enhanced under replication stress caused by HU, while the signal was gradually attenuated with the elimination of replication pressure (Figure 3E). This result implies that KDM6A cooperates with RPA proteins to protect nascent DNA during DNA replication. To further verify this possibility, we isolated the proteins on replication forks using iPOND assay. We found that the enrichment of KDM6A at replication forks was significantly increased after HU treatment and was gradually attenuated after HU removal, mimicking the process of fork restart, similarly to the dynamic of Ku70, which promotes the stability of stalled replication fork (Figure 3F). These results suggest that KDM6A is recruited to replication forks in response to replication stress. To further evaluated the impact of KDM6A on replication forks, the formation of DNA replication foci by EdU and BrdU incorporation respectively was observed under laser confocal microscope and the results showed that the number of EdU and BrdU foci in HeLa cells with KDM6A knockdown was obviously decreased compared to the control after HU treatment, indicating that the involvement of KDM6A in the regulation of replication forks formation and restart (Figure 3G). Furthermore, we labeled the nascent DNA fiber in HeLa using CldU followed by HU treatment to induce replication forks stalling. Under laser confocal microscope, we observed that the length of DNA fiber in HeLa with KDM6A knockdown was much shorter than that in the control after HU treatment, suggesting that loss of KDM6A disrupted the stability of nascent DNA (Left panel in Figure 3H). Besides, we pre-treated cells with HU to induce replication forks stalling followed by CldU incorporation to observe the restart of replication forks after washing out HU. Results revealed that the KDM6A knockdown obviously suppressed incorporation of CldU into nascent DNA, indicating that the significance of KDM6A in the restart of replication forks (right panel in Figure 3H). To further evaluate the influence of KDM6A on nascent DNA, multiple biomarkers involved in maintaining the stability of replication forks were determined by western blot. The results showed that the enrichment of H3K9ac, H4K8ac and γH2AX were significantly upregulated in HEK293T cells lacking KDM6A in response to HU (Figure 3I). In addition, the enrichment of Ku70 at the stalled replication forks were remarkably reduced in KDM6A-deficient cells detected by iPOND assay (Figure 3J). Since DSB ends including those of single ended DSBs formed upon replication fork collapse are protected by immediate loading of the Ku complex, a heterodimer composed of the Ku70 and Ku80 proteins, which stabilizes the DSB end structure and inhibits exonucleolytic degradation by EXO1 (38,39), the results in Figure 3J argue that the stability of stalled replication forks was weakened, and the stalled forks may be undergoing collapse. Taken together, these results indicate that KDM6A is recruited to nascent DNA under replication stress to maintain genomic stability through protecting replication forks from collapse.

### SND1 associates with KDM6A to regulate the genomic stability.

**Figure 4.**
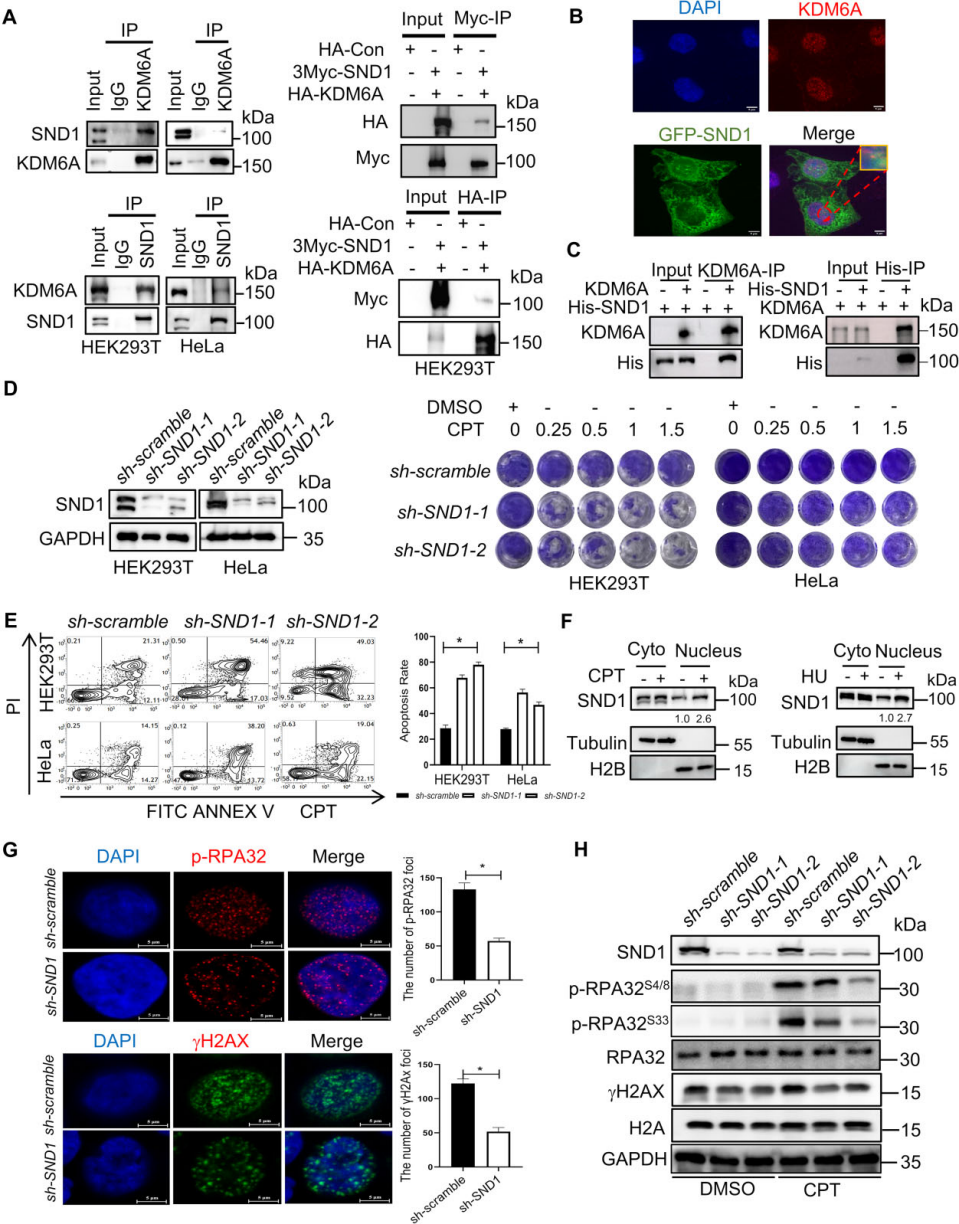
SND1 is an interactor protein of KDM6A and involved in the genomic stability. (**A**) The interaction between KDM6A and SND1 *in vivo*. KDM6A and SND1 was immunoprecipitated using specific antibodies against KDM6A and SND1 respectively or specific tag antibodies, followed by western blot with indicated antibodies. (**B**) The co-localization between KDM6A and SND1 in HeLa cells. Representative images with scale bars (5 μm) and quantifications are shown. (**C**) Detection of KDM6A–SND1 binding *in vitro* using purified recombinant proteins. *In vitro* pull-down assay was performed using purified KDM6A and His-SND1 recombinant proteins followed by detection with western blot. (**D**) The validation of SND1 knockdown by western blot (Left panel) and cellular viability against CPT in HEK293T and HeLa cells (Right panel). (**E**) Apoptosis for indicated cells with SND1 knockdown in the presence of CPT. (**F**) Western blot for the chromatin associated KDM6A and SND1. Chromatin-associated proteins were prepared from the nucleus of HEK293T cells treated with either CPT or HU and were subjected to western blot. The number represents the quantification of KDM6A against to H2B. (**G**) Image of phospho-RPA32 (S4/S8) and γH2AX foci in HeLa cells was captured by fluorescence microscopy. Representative images with scale bars (5 μm) and quantifications are shown (left). (**H**) Western blot for phospho-RPA32 (S4/S8 and S33) and γH2AX in HeLa cells. All experiments were independently repeated three times (right panel). *P < 0.05. *P* values were determined using a paired Student's *t* test.

Since KDM6A is recruited to replication forks under replication stress to promote the stability of nascent DNA independent on its histone demethylation activity, this implies that KDM6A regulates the stability of replication forks through recruiting other chromatin remodeling factors. Intriguingly, we found that staphylococcal nuclease domain-containing protein 1 (SND1) was a potential binding target of KDM6A (Figure 3A). SND1 in nucleus not only regulates gene transcription as a coactivator, but also respond to DNA damage quickly (40). To verify the interaction between KDM6A and SND1, we performed Co-IP using specific antibodies against KDM6A or SND1 respectively in HEK293T and HeLa cell. We found that KDM6A is indeed coimmunoprecipitated with SND1 (left panel in Figure 4A). Additionally, this result was further confirmed in cells over-expressing HA-KDM6A or 3Myc-SND1 by coimmunoprecipitation using specific tag antibodies (right panel in Figure 4A). Next, we performed immunofluorescence assay to investigate whether KDM6A colocalizes with SND1 in cells expressing GFP-SND1. The results showed that KDM6A foci were uniformly distributed in the nucleus, while abundant SND1 were detected in the cytoplasm. Colocalization of KDM6A with SND1 signal in the nucleus was also observed (Figure 4B). Moreover, the results from *in vitro* pull-down assay using purified proteins reveal that His-tagged SND1 indeed bind with recombinant KDM6A further supporting the interaction between KDM6A and SND1 (Figure 4C). To test the potential synergistic regulation of genomic stability by KDM6A and SND1, we first investigated the function of SND1 in cell survival and DDR. The results showed that loss of SND1 enhanced the sensitivity to DNA damaging agent CPT which is similar with the phenotype of KDM6 loss (Figure 4D–E). Moreover, the level of chromatin associated SND1 protein was also increased after HU or CPT treatment, indicating that SND1 is possibly involved in the process of DNA replication or DDR (Figure 4F). Intriguingly, absence of SND1 led to a decrease in foci formation of phospho-RPA32^S4/S8^ and γH2AX (Figure 4G), and significantly reduced the expression of phospho-RPA32^S4/S8^ and γH2AX (Figure 4H), implying the involvement of SND1 in the regulation on DDR. Considering the function of KDM6A in the regulation on genomic stability, these data suggest that interaction between KDM6A and SND1 probably plays a critical role in maintaining genomic stability.

### KDM6A interacts with the SN domain of SND1 *via* TPR and JmjC domains

**Figure 5.**
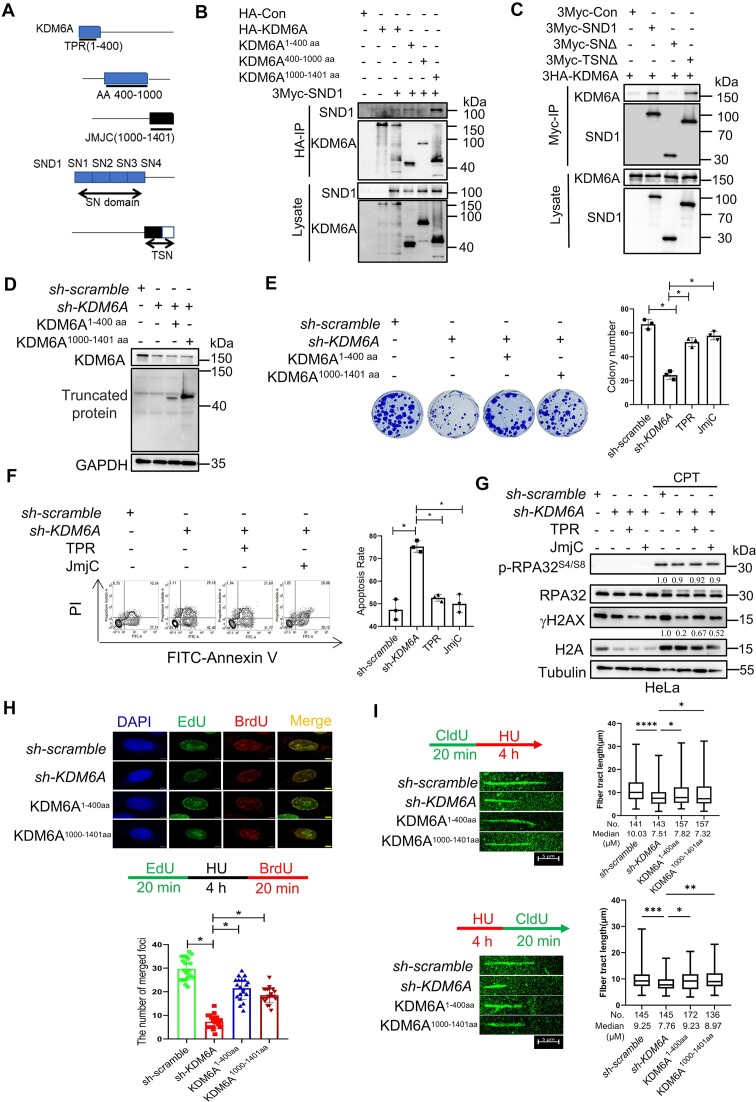
The TPR-JmjC domain of KDM6A interacted with SN domain of SND1 and involved in the stability and restart of replication forks. (**A**) Schematic diagram shows the construction strategy of KDM6A and SND1 mutants. (B, C) The domain of TPR and JmjC in KDM6A and SN domain in SND1 contribute to KDM6A–SND1 interaction. After co-transfection of KDM6A and SND1, KDM6A (**B**) or SND1 (**C**) was immunoprecipitated in HEK293T cells using specific tag antibodies, followed by western blot with indicated antibodies. (**D–F**) Complement of KDM6A TPR or JmjC domain partially rescue the viability of HeLa cells lacking endogenous KDM6A in response to CPT treatment. (**G**) Western blot for phospho-RPA32^S4/S8^ and γH2AX in HeLa cells. Lysate from cells lacking endogenous KDM6A in combination of KDM6A TPR or JmjC domain complement was subjected to western blot. Quantification of phospho-RPA32^S4/S8^ and γH2AX protein were determined against RPA32 and γH2AX expression respectively. (**H**) BrdU and EdU were incorporated into nascent DNA for immunofluorescence observation. The histogram showed the number of replication foci formation in indicated cells. (**I**) DNA fiber assay for exploring the influence of KDM6A on the progression (upper panel) and restart (lower panel) of DNA replication forks. The histogram showed the alteration in the length of nascent DNA in HeLa. All experiments were independently repeated three times, *P < 0.05. *P* values were determined using a paired Student's *t* test.

The above findings have revealed that KDM6A is not only involved in maintaining the genomic stability, but also interact with SND1, implying a potential coordinated role of KDM6A and SND1 in regulating the genomic stability. However, the mechanism behind this process remains unclear. To address this question, we first mapped the domain of KDM6A responsible for SND1 binding. TPR and JmjC are the classic functional domains of KDM6A. It has been found that the TPR domain of KDM6A mainly mediates PPI while JmjC domain is mainly responsible for histone demethylation. Additionally, intrinsic disorder sequence between TPR and JmjC domains regulates the distribution of KDM6A on chromatin through phase separation and play an important role in tumor suppression. SND1 comprises tandem repeats of four nuclease domains (SN) and a fifth domain containing fusion of Tudor and partial nuclease domains (TSN). To identify the motif mediating the interaction between KDM6A and SND1, we constructed the truncated KDM6A and SND1 plasmids respectively (Figure 5A). Co-immunoprecipitation showed that the 1–400 aa and 1000–1401 aa of KDM6A were able to capture SND1 but the 400–1000 aa did not, suggesting that TPR and JmjC are key domains mediating this interaction (Figure 5B). Moreover, SN domain, instead of TSN of SND1 domains efficiently co-precipitated with KDM6A, suggesting that the interaction between SND1 and KDM6A is mainly mediated by SN domain (Figure 5C). To further elucidate the role of TPR and JmjC domains, plasmids encoding these domains were transfected into KDM6A-deficient HeLa cells. The expression of the two domains was verified by western blot using specific antibody against HA-tag (Figure 5D). In the presence of CPT, we found that the cell survival was partially recovered by TPR or JmjC domains, indicating the importance of TPR and JmjC domains are important for cell growth. (Figure 5E and F). In addition, overexpression of TPR or JmjC domains rescued the expression of γH2AX in KDM6A-deficient HeLa cells, suggesting the potential involvement of TPR and JmjC domains in the activation of ATR or ATM pathway (Figure 5G). To assess the effect of TPR and JmjC domains on the stability and restart of replication forks, we employed BrdU/EdU dual labeling assay and DNA fiber assay. The results showed that TPR or JmjC domain not only obviously restored the formation of BrdU and EdU foci (Figure 5H), but also the length of nascent DNA fiber before (Upper panel in Figure 5I) or after (Upper panel in Figure 5I) HU treatment, suggesting their pivotal role in regulating replication forks stability and replication restart. Together, these data suggest that TPR-JmjC domain is required for the formation of SND1-KDM6A complex and the regulation of replication forks stability.

### KDM6A–SND1 complex maintains replication forks stability through protecting the nascent DNA from degradation in coordination with RPA1 and Ku70

**Figure 6.**
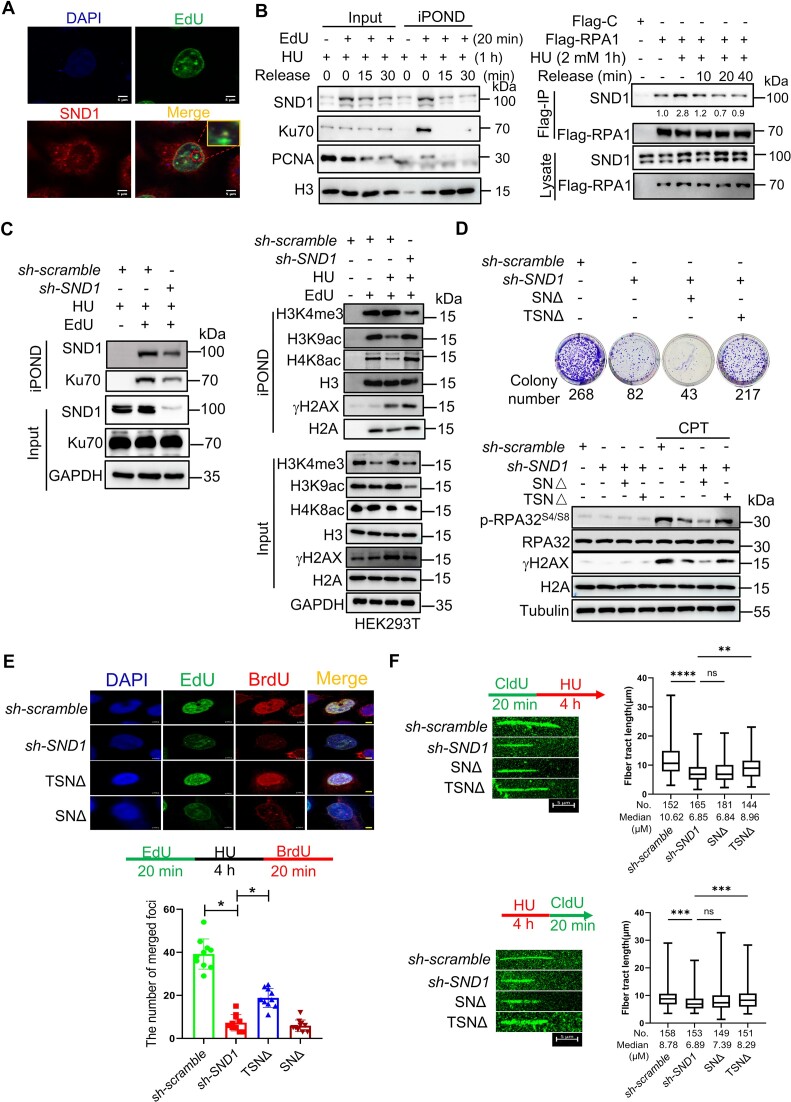
SN domain mediated the regulation of stability and restart of replication forks by SND1. (**A**) The association of SND1 with nascent DNA in HeLa cells treated with CPT. EdU incorporation was performed as previously publications. Representative images with scale bars (5 μm) are shown. (**B**) Western blot of nascent DNA-associated proteins at stalled replication forks. Nascent DNA-associated proteins were isolated in HEK293T cells treated with HU for 1 h and followed by releasing into fresh media for indicated time using iPOND approach, and the resulted proteins were detected using specific indicated antibodies (left panel). Western blot of RPA1 immunoprecipitation in HEK293T cells treated with HU for 1 hour and followed by releasing into fresh media for indicated time. Copurified proteins were detected using specific antibodies. The number represented the quantification of SND1 protein against to Flag-RPA1 (right panel). (**C**) Western blot for H3K9ac, H4K8ac, γH2AX and Ku70 at the nascent DNA in SND1 deficient HEK293T cells. (**D**) Complement of SN domain of SND1partially rescue the viability of HeLa cells lacking endogenous SND1 protein in response to CPT treatment (Upper panel). Western blot for phospho-RPA32^S4/S8^ and γH2AX in HeLa cells in response to CPT treatment (Lower panel). (**E**) BrdU and EdU were incorporated into nascent DNA for observing the replication forks foci formation and restart of replication forks by immunofluorescence method. The histogram showed the number of replication foci formation in indicated HeLa cells. Scale bars, 5 μm. (**F**) DNA fiber assay for exploring the function of SND1 and its truncated protein on the progression (upper panel) and restart (lower panel) of DNA replication forks. The histogram showed the alteration in the length of nascent DNA in HeLa. All experiments were independently repeated three times, *P < 0.05.

To further investigate the role of KDM6A–SND1 complex in regulating replication forks stability, we first evaluated the effect of SND1 on nascent DNA. To this end, EdU was incorporated into nascent DNA of HeLa cells followed by HU treatment and the co-localization signal between SND1 and EdU was captured by laser confocal microscope. The fluorescence image clearly depicted the colocalization of SND1 with EdU-labeled nascent DNA, suggesting that SND1 is indeed enriched on nascent DNA under replication stress (Figure 6A). To further confirm this result, we utilized iPOND assay to detect the protein on stalled replication forks and found that the enrichment of SND1 at replication forks in HEK293T was obviously increased after HU treatment, which was decreased upon HU removal (left panel in Figure 6B). Moreover, the interaction between SND1 and Flag-RPA1 was also enhanced in the presence of HU and attenuated following HU withdraw (right panel in Figure 6B). This trend was similar to that observed for KDM6A detected by iPOND (Figure 3E). In addition, we also detected multiple biomarkers related to the stability of the nascent DNA and found that the level of H3K9ac, H4K8ac and γH2AX on nascent DNA was significantly upregulated in SND1-deficient HEK293T cells in response to HU. Conversely, the level of Ku70 at the stalled replication forks were remarkably reduced, suggesting that recruitment of SND1 at replication forks promoted the stability of nascent DNA through Ku70 recruitment and histone modification (Figure 6C). Interestingly, we found that exogenous SN domain (TSNΔ) could restore the ability of colony formation and the expression of phosphor-RPA32^S4/S8^ and γH2AX, indicating that SN domain of SND1 plays an important role in genomic stability (Figure 6D). Furthermore, the formation of BrdU and EdU foci in HeLa cells lacking SND1 after HU treatment showed obvious suppression compared to negative control (Figure 6E). Consistent with the results of colony formation and the expression of phosphor-RPA32^S4/S8^ and γH2AX, SN domain could partially restore the formation of BrdU and EdU foci (Figure 6E). Additionally, the results of DNA fiber assay showed that loss of SND1 not only disrupt the stability of nascent DNA (upper panel in Figure 6F) but also suppress the restart of replication forks, while exogenous SN domain expression partially restores nascent DNA fiber length and replication restart (lower panel in Figure 6F), implying that SN domain is required for maintaining the stability of replication forks. These data indicate that SND1 contributes substantially to the stability and restart of replication forks probably *via* SN domain.

Our data showed that KDM6A and SND1 knockdown both impaired the Ku70 enrichment at nascent DNA. To further investigate the mechanism, we performed co-IP to study the interaction of Ku70 with KDM6A–SND1 complex. Our results revealed that KDM6A could interact with Ku70, while SND1 could not. This interaction could be strengthened by CPT treatment (Supplementary Figure S2A). The co-localization signal of KDM6A and Ku70 was also observed successfully in HeLa and obviously enhanced by CPT (Supplementary Figure S2B), indicating that Ku70 at nascent DNA could be recruited by KDM6A in response to replication stress. Notably, we did not observe the interaction between SND1 either with Ku70 or PCNA using Co-IP assay, though PCNA was detected in our mass spectrometry data (Supplementary Figure S2C). Moreover, we screened the key domain mediated the interaction of KDM6A with Ku70 and found that TPR domain was critical for their interaction (Supplementary Figure S2D). Furthermore, the iPOND results revealed that KDM6A enrichment at replication forks after HU treatment was almost abolished in SND1-deficient HEK293T cells and could be restored by exogenous SND1 and TSNΔ protein (Supplementary Figure S2E). Based on the results of *in vivo* Co-IP and *in vivo* iPOND assays, we could conclude the interaction between KDM6A and SND1 is indeed involved in recruiting Ku70 onto nascent DNA to maintain fork stability. Thus, this interaction seems act as a scaffold mediating Ku70 recruitment onto nascent DNA under replication stress. However, we could not rule out the possibility which other factors may be involved this process.

### KDM6A–SND1 interaction is up-regulated by KDM6A SUMOylation

**Figure 7.**
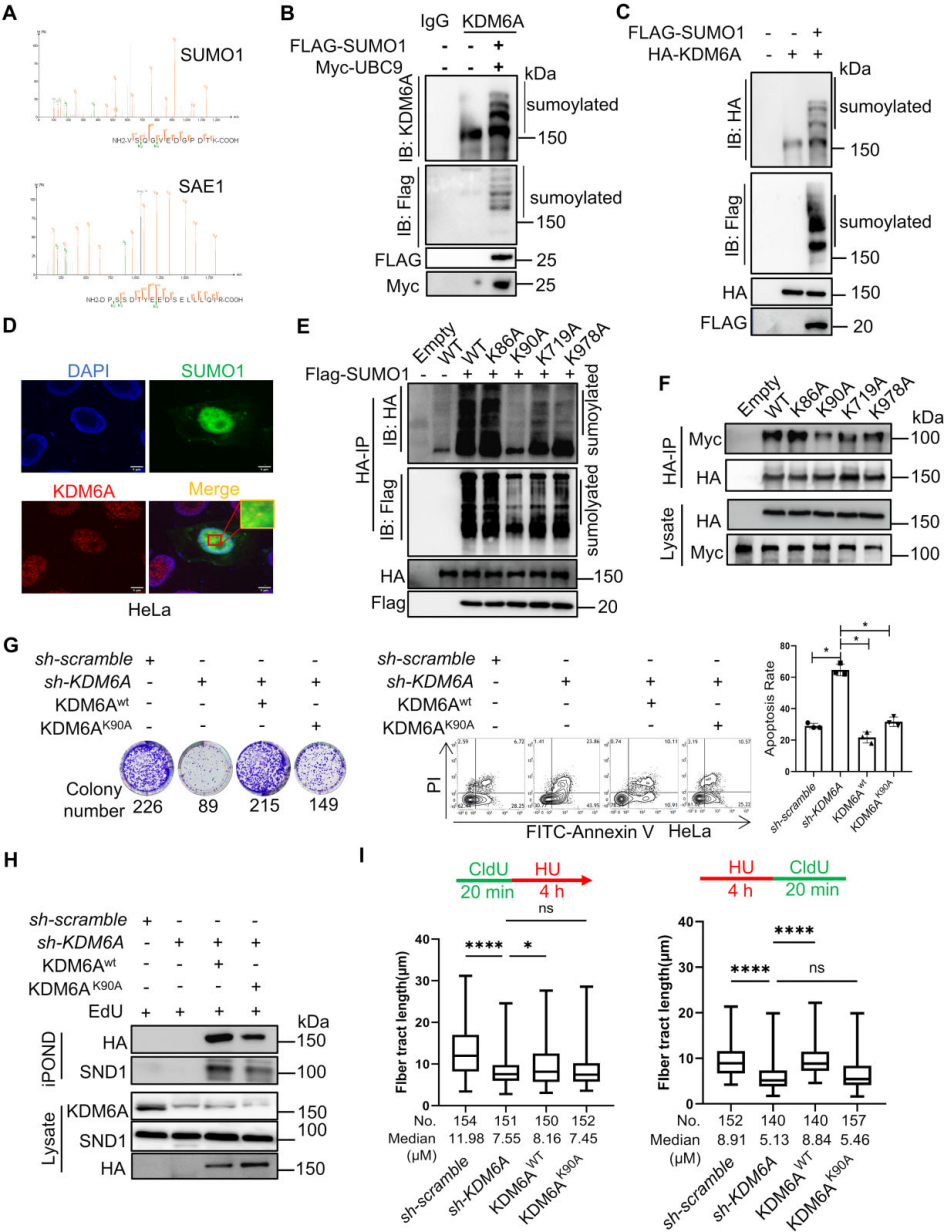
KDM6A SUMOylation increases KDM6A–SND1 interaction and involved in the stability and restart of DNA replication forks. (**A**) SUMO1 and SAE1 were identified by mass spectrometry in KDM6A coimmunoprecipitation samples. (B, C) Detection of KDM6A SUMOylation by Co-IP followed by western blot with indicated antibodies. Co-IP was performed in HEK293T cells transfected with either Flag-SUMO1 alone (**B**) or together with KDM6A plasmids (**C**). SUMOylation of endogenous (B) and exogenous KDM6A (C). (**D**) The co-localization between KDM6A and SUMO1 in cells. Representative images with scale bars (5 μm) and quantifications are shown. (**E**) SUMOylation of KDM6A mutants. co-IP was performed in HEK293T cells transfected with Flag-SUMO1 and KDM6A plasmids and followed by western blot with indicated antibodies. (**F**) The interaction between KDM6A mutant(s) and SND1. Co-IP was performed in HEK293T cells transfected with the plasmid of HA-KDM6A and Myc-SND1, followed by western blot with indicated specific antibodies against tag. (**G**) Complement of KDM6A^K90A^ partially restore the colony formation (left) and survival (right) of HeLa cells lacking endogenous KDM6A protein in response to CPT. (**H**) The enrichment of SND1, KDM6A^K90A^, KDM6A^wt^ on nascent DNA was determined by iPOND and western blot. (**I**) The histogram represented the restoration in length of DNA fiber in HeLa cells lacking endogenous KDM6A with KDM6A^K90A^ and KDM6A^wt^ complement respectively. All experiments were independently repeated three times, *P < 0.05.

The PPI is regulated not only by protein domain, but also by protein post-translational modifications (PTMs). SUMOylation is one of the PTMs, which often occurs at the lysine residues of proteins, and it is involved in the regulation of numerous physiological events, including gene transcription, DNA replication, DDR and RNA processing through modulating protein interaction, subcellular localization, protein degradation and others (41). Our LC–MS data revealed that both SUMO1 and SAE1 were coimmunoprecipitated with KDM6A, implying a possibility that KDM6A might be modified by SUMO1 (Figure 7A). To functionally validate this possibility functionally, co-IP was performed to determine the covalent modification between endogenous KDM6A and SUMO1. The results clearly showed that multiple protein bands were detected by western blot in co-precipitated sample of HEK293T cells (Figure 7B), suggesting that SUMOylation might occur on KDM6A. Therefore, we further detected KDM6A SUMOylation in 293T cells co-transfected with the HA-tagged KDM6A and Flag-tagged SUMO1 expression plasmids. The results showed that multiple upward-shifted protein bands of KDM6A were detected using specific antibodies against HA or Flag tag, further support the notion that SUMOylation indeed occur in KDM6A protein (Figure 7C). In addition, we also observed the co-localization of KDM6A with SUMO1 in cells, providing additional support for KDM6A SUMOylation (Figure 7D). Generally, SUMO protein is covalent connected to the lysine residue of target protein(s), which confers new functions or leads to protein degradation of the target protein. To explore the function of KDM6A SUMOylation, we analyzed the potential SUMO modification sites of KDM6A using an online database and found that K86, K90, K719 and K978 were potential SUMOylation sites (Supplementary Figure S3A). To validate this, we conducted *in vivo* SUMO assay in cells transfected with HA-tagged KDM6A harboring K86A, K90A, K719A and K978A mutations. The results clearly showed that K90A mutation almost eliminated KDM6A SUMOylation compared to the wild-type (Figure 7E). Moreover, K719A and K978A mutants also showed the reduction in KDM6A SUMOylation, although to a lesser extent than that in K90A mutant. However, K86A had no effect on the SUMOylation level of KDM6A (Figure 7E). In addition, the mutation in KDM6A SUMOylation sites, including K90A, K719A and K978A remarkably attenuated the interaction between SND1 and KDM6A, suggesting the involvement of SUMOylation in the formation of KDM6A–SND1 complex (Figure 7F). To further explore the influence of SUMOylated KDM6A on genomic stability, the wild-type and K90A mutation of KDM6A were complemented, respectively. Intriguingly, the ability of colony formation was obviously enhanced by complementing wild-type KDM6A, but the K90A mutant failed. In addition, the result of apoptosis also supported the discovery from colony formation (Figure 7G). These results imply that SUMOylation of KDM6A plays an important role in maintaining genomic integrity. Moreover, iPOND results revealed that the enrichment of KDM6A at replication forks was impaired under replication stress in K90A mutant cells (Figure 7H). Furthermore, the recruitment of SND1 at replication forks was also decreased in K90A mutant cells (Figure 7H). Similarly, while the formation of replication foci and the length of nascent DNA were partially restored by K90A complement in cells lacking KDM6A but were almost completely restored by wild-type KDM6A complementation (Figure 7I and Supplementary Figure S3B). Together, these data indicate that KDM6A SUMOylation probably not only enhances its interaction with SND1, but also promotes the recruitment of KDM6A–SND1 complex at the stalled replication fork to protect the fork from collapse.

### KDM6A promotes chemoresistance in esophageal squamous cell carcinoma

**Figure 8.**
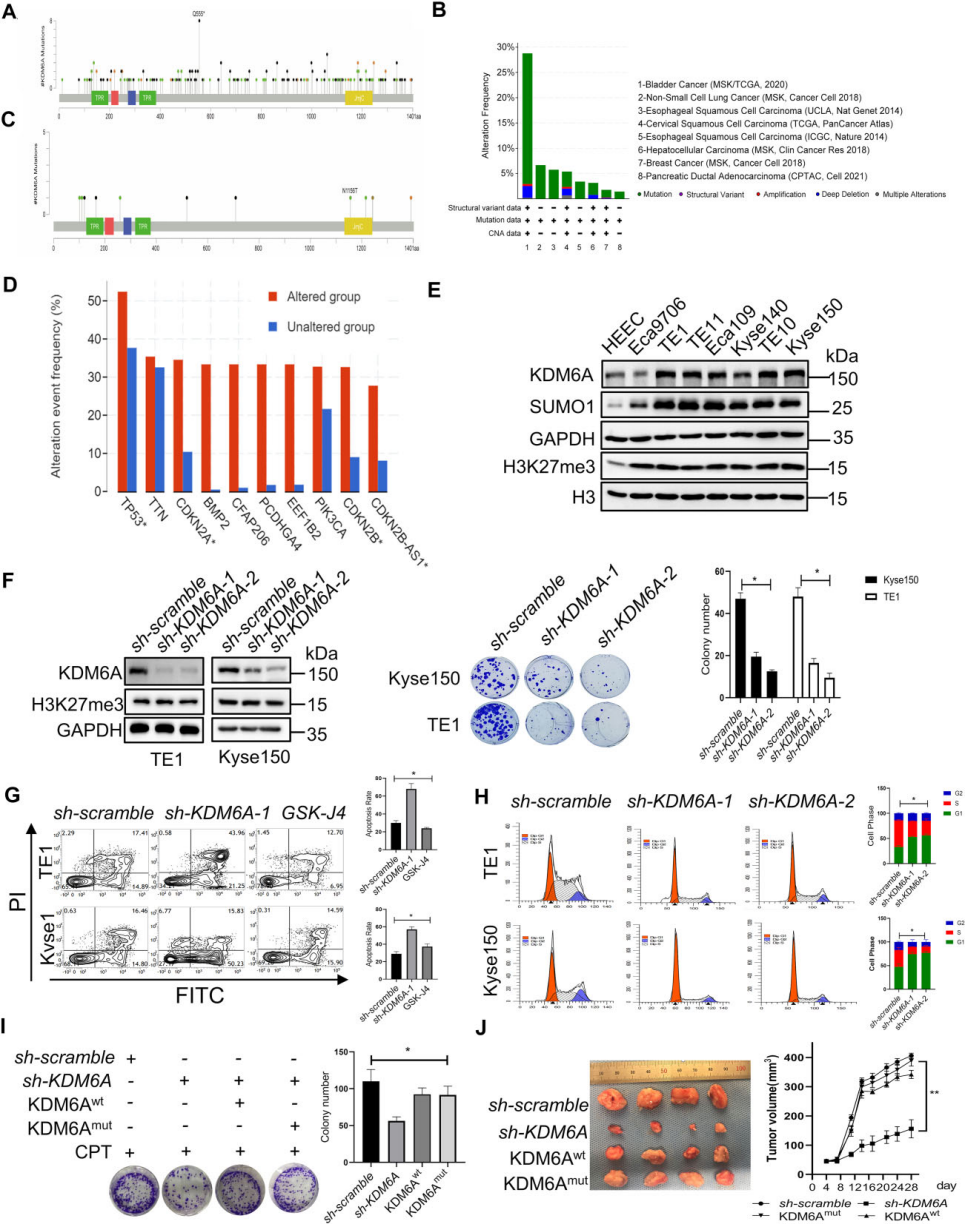
Loss of KDM6A increases the sensitivity of ESCC toward CPT. (**A–D**) KDM6A mutations and expression in tumors was analyzed using data from public database Cbioportal. (**E**) Western blot of KDM6A expression in ESCC cells. The lysate was prepared from ESCC cells including Eca9706, Eca109, TE1, TE10, TE11, Kyse140, Kyse150 and was subjected to western blot with indicated antibodies. (**F**) Cellular viability for indicated ESCC cells with KDM6A knockdown in the presence of genotoxin CPT (Middle panel). Quantification of colony formation was shown (Right panel). KDM6A knockdown was validated by western blot (Left panel). (G, H) Apoptosis of ESCC cells lacking KDM6A in response to CPT (**G**) and cell cycle profile of ESCC cells without CPT treatment (**H**). (**I**) Cellular viability for ESCC cells knocked down endogenous KDM6A and followed by transfection with the plasmid of KDM6A^wt^ or catalytic-dead KDM6A^mut^ respectively. Quantification of colony formation was shown (Right panel). (**J**) Inhibition of tumorigenesis *in vivo* by knocking KDM6A down. Xenograft was generated using ESCC cells same as it in panel H (left panel), and tumor size was measured at the end of the study (right panel). Data represent mean ± S.E.. All experiments were repeated for three times independently, *P < 0.05. **P < 0.01.

Recent investigations into genomic stability maintenance have revealed previously hidden layers of drug resistance that depend on the dysregulation of genome stability in tumor. We have shown that KDM6A is recruited at the stalled replication forks to protect nascent DNA in coordination with RPA1 and Ku70 proteins, suggesting a possible hotspot for overcoming drug resistance of tumors through KDM6A. To investigate the effect of KDM6A on drug resistance, we firstly analyzed the genetic characteristics of KDM6A associated with tumors using online database Cbioportal (www.cbioportal.org) and timer2.0 (TIMER2.0 comp-genomics.org). The results revealed the aberrant expression of KDM6A in multiple tumors and the mutations of KDM6A in tumors were widely distributed in the TPR, JmjC domain, and disordered sequence region. These findings implied that these mutations might impair KDM6A functions including PPI (Figure 8A). Intriguingly, we also noticed that the expression of KDM6A remained unchanged in esophageal squamous cell carcinoma (ESCC) and glioblastoma (GBM) in comparison with para-cancerous tissues (Figure 8A and Supplementary Figure S4A, S4B). Moreover, several mutations of KDM6A occurred in ESCC and the mutational frequency was only up to 5% (Figure 8B, C). Correlation analysis showed a positive correlation between the mutational frequency of genomic stability-related genes including TP53, CDKN2A/2B, PCDHGA4, CFAP206 and EEF1B2 was positively correlated with KDM6A mutation, further supporting that KDM6A dysfunction is closely related to genomic instability in tumor (Figure 8D). Esophageal cancer is a malignant tumor with a high mortality and incidence worldwide (42,43). There is convincing evidence showing that chemoresistance stands one of the eventual reasons that leading to the failure of esophageal tumors treatment (44). Therefore, we delved deeper into the impact of KDM6A on the chemoresistance of ESCC. In line with recent discovery indicating a significant upregulation of H3K27me3 expression in ESCC tissues is significantly upregulated (45,46), we found an obviously upregulation of KDM6A expression in Kyse150, TE1 and TE10 ESCC cells while it is downregulated in Eca9706 cells compared to HEEC (Figure 8E). However, H3K27me3 expression was significantly upregulated in all ESCC cell lines tested (Figure 8E). Thus, it seems that the elevated expression of KDM6A in ESCC was not closely related to H3K27me3 expression. Interestingly, the alteration of H3K27me3 expression was undetectable in cells lacking KDM6A, suggesting that KDM6A probably is not a major contributor for H3K27me3 demethylation in ESCC cells (Figure 8F). In addition, KDM6A knockdown inhibited the colony formation and increased apoptosis in the presence of CPT, suggesting that KDM6A elimination promotes the sensitivity to genotoxin for ESCC (Figure 8F and G). Intriguingly, GSK-J4, a KDM6A inhibitor, failed to boost the apoptosis toward CPT treatment, implying that KDM6A activity is dispensable in ESCC (Figure 8G). However, loss of KDM6A obviously arrested cell cycle at G1 phase (Figure 8H), further supporting the involvement of KDM6A in regulation on DNA replication. To further confirm that KDM6A functions in genomic stability independently of its demethylation activity, we investigated cell viability of ESCC cells in response to CPT treatment. First, we knocked down endogenous KDM6A and then complemented with with KDM6A^wt^ or catalytic-dead KDM6A^mut^. The results showed that both KDM6A^wt^ and KDM6A^mut^ has comparable effect on rescuing cell growth in response to CPT treatment (Figure 8I). The similar results were also observed in xenograft model generated with ESCC cells expressing KDM6A^wt^ or KDM6A^mut^ respectively (Figure 8J). Altogether, these results suggest that abnormally high expression of KDM6A substantially contributes to the chemoresistance of ESCC in a manner independent of its enzyme activity.

### KDM6A–SND1 interaction enhances chemoresistance in ESCC

**Figure 9.**
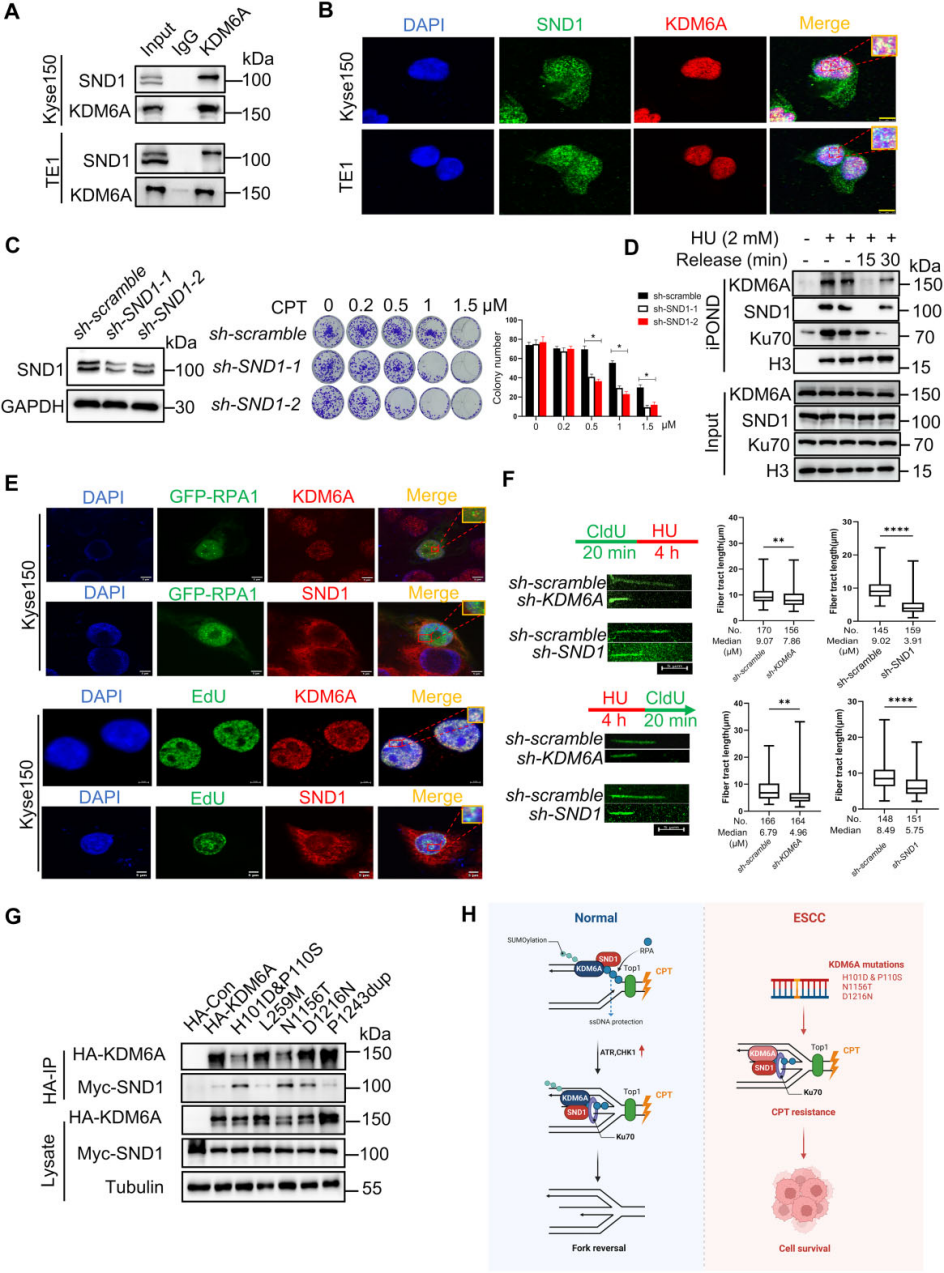
KDM6A and SND1 synergistically promotes the resistance of ESCC to CPT. (A-B) The interaction (**A**) and co-localization (**B**) of KDM6A–SND1 in in Kyse150 and TE1 cells was shown. Scale bars, 5 μm. (**C**) Cellular viability for ESCC Kyse150 cells with SND1 knockdown in the presence of genotoxin CPT. (**D**) Western blot of nascent DNA associated proteins at stalled replication forks. Nascent DNA-associated proteins were isolated in Kyse150 cells treated with HU for 1 h and followed by releasing into fresh media for indicated time using iPOND approach, and the resulted proteins were detected using specific indicated antibodies. (**E**) The association of KDM6A or SND1 with EdU and GFP-RPA2 in Kyse150 cells treated with CPT. Representative images with scale bars (5 μm) are shown. (**F**) The length of nascent DNA fibers was evaluated by DNA fiber assay. New incorporated CldU (green) into nascent DNA followed by HU treatment determined the stability of replication forks (upper panel), and the incorporated CldU in nascent DNA after HU treatment was used to evaluate the restart of replication forks (lower panel). (**G**) Co-IP assay was performed in Kyse150 cells co-transfected the plasmid of SND1 and KDM6A mutants identified in ESCC patients and followed by western blot using specific antibodies. (**H**) Schematic diagram of the underlying mechanism by which KDM6A–SND1 regulates the genomic stability and ESCC chemosensitivity. Alterations of KDM6A directly regulates the interaction with SND1, leading to subsequent the recruitment of Ku70 and RPA proteins to replication fork to prevent nascent DNA from degradation, eventually affect genotoxin drug chemosensitivity in cancer. **P < 0.01. ****P < 0.0001.

To investigate the possible role of KDM6A–SND1 interaction in ESCC cells, we first employed co-immunoprecipitation assay using KDM6A antibody and confirmed their interaction (Figure 9A). Consistent with our Co-IP results, we also observed the colocalization of KDM6A and SND1, supporting that the interaction capability of KDM6A with SND1 in ESCC (Figure 9B). Given our demonstration of the critical role of KDM6A–SND1 interaction in regulating the stability of DNA replication fork, and the contribution of KDM6A to chemoresistance in an enzyme activity-independent manner, we hypothesize that SND1 is likely involved in chemoresistance as well. Indeed, we observed the substantially increased sensitivity to CPT in ESCC cells lacking SND1 expression (Figure 9C), suggesting that KDM6A–SND1 probably regulate drug sensitivity in a coordinated manner. Moreover, we confirmed the recruitment of KDM6A, SND1 and Ku70 at stalled replication forks detected by iPOND. The release of these proteins from fork upon replication restart, and the colocalization of KDM6A/SND1 with RPA1 or EdU in ESCC cells, implying that KDM6A and SND1 are required for ESCC cell survival under the stress caused by genotoxin (Figure 9D, E). Additionally, we also evaluated the impact of KDM6A and SND1 on nascent DNA in ESCC using DNA fiber assay. We confirmed that the length of DNA fiber in ESCC cells lacking KDM6A or SND1 after HU treatment was significantly decreased compared to the control group (upper panel in Figure 9F). Similarly, the nascent DNA in indicated ESCC cells pre-treated with HU was obviously shorter than that in control group (lower panel in Figure 9F). These data suggest that KDM6A and SND1 play a pivotal role in the stability and restart of replication forks in ESCC. Next, we investigated whether there are functional mutations of KDM6A in ESCC patients. By analyzing the public whole-genome sequencing data, we identified five mutations in KDM6A including H101D & P110S, L259M, N1156T, D1216N, P1243dup identified in ESCC patients. We argue that these mutations may affect its interaction capability with SND1. To test this possibility, we performed Co-IP assay, and found that KDM6A H101D & P110S, N1156T and D1216N mutants indeed increase the interaction with SND1 as expected, but not L259M and P1243dup (Figure 9G). To further examine the influence of KDM6A mutants on chemoresistance of ESCC, we performed colony formation experiment in KDM6A deficient cells either expressing wild-type KDM6A or these mutants. The results showed that the sensitivity toward CPT was substantially attenuated in cells harboring either H101D & P110S, N1156T or D1216N mutation respectively (Supplementary Figure S5A). Meanwhile, the level of phospho-RPA32^S4/8^ and γH2AX were obviously increased in cells harboring either H101D & P110S, N1156T or D1216N mutation respectively, compared to that in cells lacking KDM6A (Supplementary Figure S5B). The above results suggest abnormal KDM6A–SND1 interaction possibly contribute to the chemoresistance. Taken together, except with known functions of KDM6A and SND1, these findings unambiguously demonstrate that KDM6A–SND1 interaction is required for the stability of replication forks and the chemoresistance in ESCC cells.

## Discussion

Genomic stability mainly refers to the precise and orderly execution of DNA synthesis, replication, gene transcription, nucleosome assembly and mitosis. These processes are closely related to the formation of protein complex, histone modifications, post-translational modifications (PTMs) and other factors (47,48). Moreover, restoration of genome integrity is associated with chemoresistance of tumors (49–51). While multiple chromatin modifying factors such as EZH2, HAT1, KDM6B and TET2 have been confirmed to protect genome integrity, the direct involvement of KDM6A in maintaining genomic stability remains unclear. Here, we demonstrated that KDM6A regulates genomic stability independent of its demethylation activity. Moreover, we revealed that KDM6A promotes genomic stability through binding to SND1 via its TPR and JmjC domain, and the SUMOylation on KDM6A enhances its interaction with SND1. Mechanistically, KDM6A and SND1 are recruited to stalled replication forks induced by genotoxin HU to facilitate the enrichment of Ku70 and H3K4me3 on nascent DNA and suppresses the enrichment of H3K9ac and H4K9ac, eventually protect the replication fork from collapse. In contrast, depletion of KDM6A or SND1 in cells represses the enrichment of Ku70 and H3K4me3 on nascent DNA but increase the enrichment of H3K9ac and H4K8ac, leading to the inhibition of replication forks restart and the genomic instability. Interestingly, ESCC cells lacking KDM6A or SND1 exhibit increased sensitive to genotoxin CPT. Moreover, mutations in KDM6A, such as H101D & P110S, N1156T and D1216N identified in ESCC patients increase its interaction capability with SND1 and confer resistance to genotoxin resistance. Conversely, knockdown of KDM6A and SND1 in ESCC cells attenuates genotoxin resistance. These findings potentially explain why loss of KDM6A promotes genomic instability in ESCC cells and renders them chemosensitivity. Our works suggest that KDM6A–SND1 may impact the response to genotoxic chemotherapy and shed light on the potential clinical implications.

KDM6A is known as one of the H3K27me3 demethylases and plays a critical role in transcription, embryo development and tumorigenesis (52,53), the biological function of KDM6A in genomic stability so far has not been well investigated thus far. Genomic instability, recognized as one of cancer hallmarks, contributes to tumorigenesis and serves as the basis for developing therapeutic approaches. Previous studies have suggested that the demethylation of H3K27me3 is involved in regulation of genomic stability. Additionally, reduced H3K27me2/me3 reduction caused by H3.1K27M mutation suppressed the formation of 53BP1 foci and the capability of non-homologous end joining (NHEJ) in diffuse intrinsic pontine glioma (54,55). Despite all this, KDM6A as one of H3K27me3 demethylase, using both pharmacological KDM6A inhibition and genetic KDM6A catalytic mutant inactivation, we show that KDM6A catalytic activity is not required for regulating genomic stability. This difference suggests that KDM6A may form specific complex(es) in the different context. Given that KDM6A also function as a component of multiple protein complex instead of solely histone demethylase, for instance, KDM6A could form the COMPASS complex with MLL3, RBBP5, WDR5, and DPY30 mediated by TPR domain, thereby regulating histones H3K4me and H3K27ac level on chromatin (56,57). Moreover, besides the COMPASS complex, KDM6A could bind to other small molecular proteins, such as PTIP and SPT6, as demonstrated by exclusion chromatography (58). Indeed, we have identified SND1 as one of KDM6A associated proteins. SND1, a multifunctional protein distributed widely in both cytoplasm and nucleus (59,60), not only participates in the formation of stress granules, but also regulates gene transcription through recognizing histone methylation modifications (61,62). Recent works have demonstrated that SND1 is recruited to DSB sites to promote DNA repair via recruiting GCN5 and SMARCA5, indicating that SND1 its role in maintaining genomic stability (63). As we expected, SND1 indeed is required for cell viability in response to genotoxin CPT, indicating the involvement of KDM6A–SND1 complex in maintaining genomic stability.

Stalled replication forks induced by replication stress constitute a pivotal factor leading to genomic instability (64). When replication forks stall, nascent DNA undergoes reversion to form the ‘Holliday junction’ structure when replication fork stopped (65). The regulation of stalled replication forks involves in a complex interplay of histone modifications and chromatin remodeling factors, resembling the process of DNA repair on chromatin. For example, PTIP, an interactor of KDM6A, promotes the recruitment of the MRE11 nuclease to stalled replication forks to prevent extensive degradation of nascent DNA (66). Moreover, H3K27me3 regulated by EZH2 at stalled replication forks can recruit MUS81 and cause degradation of the forks (67). Intriguingly, loss of KDM6A could induce the G1/S arrest. As well known, the activation of MCM2–7 complex at replication origins by cyclin-dependent kinase 2 (CDK2) promotes the assemble of replication forks when cells enter S phase. Aberrant replication forks could induce replication stress subsequently resulting in G1/S arrest. As shown in Figure2D, there are some differences in cell cycle between wild-type and catalytic-dead KDM6A. Thus, it is worth of further exploration to the role of wild-type and catalytic-dead KDM6A in cell cycle profile. Together, these findings support that KDM6A is probably involved in stabilizing replication forks. Consistent with the findings that KDM6A has a profound effect on the prevention of nascent DNA degradation at stalled forks, both KDM6A and SND1 are recruited to stalled replication forks as determined by iPOND assay (Figure 6F and Figure 8F). RPA1 participates in replication fork stability by coating single DNA strand quickly to protect the nascent DNA from degradation at stalled forks (68,69). Interestingly, it is observed that the interaction between RPA1 and KDM6A–SND1 complex is significantly enhanced under the replication stress (Figure 6E).

The mechanisms underlying the maintenance of stalled replication fork stability involve the coordinated actions of multiple proteins on nascent DNA. For instance, Ku70 exerts critical function in forks stability and could quickly recognize and bind to the end of reversed DNA double strand in the presence of replication stress (70,71). Our findings are consistent with the role of Ku70 as a protector for nascent DNA. Specifically, we demonstrated that knockdown of either KDM6A or SND1 significantly suppresses the enrichment of Ku70 on nascent DNA while increased the level of γH2AX, implying the occurrence of DNA damage on nascent DNA. In addition to either single protein, our study highlights the involvement of KDM6A–SND1 interaction in recruiting Ku70 onto nascent DNA for maintaining fork stability. This interaction appears to act as a scaffold mediating Ku70 recruitment to nascent DNA under replication stress, though other factors may also play a role in this process. Notably, we did not observe any interaction between KDM6A/SND1 and PCNA, despite the critical role of Rad18-mediated PCNA monoubiquitylation by ATR activation and SLX4 play a critical role in preserving replication fork stability (72). Moreover, we further show that knockdown KDM6A or SND1 leads to a reduction in H3K4me3 enrichment but an increase in H3K9ac and H4K8ac on nascent DNA. The presence of H3K4me3 at stalled forks is known to protect the nascent DNA from degradation by nuclease (73). On the contrary, H4K8ac promotes the nascent DNA degradation by recruiting EXO1 and MRE11 (74). Thus, it is conceivable that the reduced enrichment of Ku70 and H3K4me3, coupled with the increased enrichment of γH2AX, H3K9ac and H3K8ac on nascent DNA in cells lacking KDM6A or SND1 is a predisposition to the degradation of nascent DNA. This conclusion is further confirmed by DNA fibers spreading assay. When evaluating the stability or restart of stalled replication forks, the length of DNA fiber obtained from cells with either KDM6A or SND1 depletion are obviously shorter than negative control, indicating the involvement of KDM6A and SND1 in the stabilization and restart of replication forks. Moreover, the TPR and JmjC domains of KDM6A mediate its interaction with the SN domain of SND1, safeguarding nascent DNA from degradation and promote the restart of stalled replication forks. Intriguingly, KDM6A could undergo SUMOylation by SUMO1. Emerging evidence reveal that SUMOylation plays an important role in the protein-protein interaction. Noteworthy findings are that the SUMOylation at lysine 90 of KDM6A promotes its interaction with SND1. Conversely, the K90A mutation in KDM6A leads to instability and impedes the restart of replication forks, resulting in genomic instability. Additionally, the recruitment of KDM6A bearing the K90A mutation to stalled forks is attenuated compared to wild-type KDM6A. Subsequently, the enrichment of SND1 at stalled replication forks is also decreased, indicating that the interaction between KDM6A and SND1 regulates replication fork stability. Together, our findings support a coherent model in which KDM6A is coordinated with SND1 to modulate DNA replication fork stability.

Despite the effectiveness of intensive chemotherapy for initial treatment, relapse due to intrinsic or acquired the resistance poses a major challenge. Numerous studies proved compelling evidence indicating that KDM6A in tumor cells not only contributes to tumorigenesis but also increases resistance to chemotherapy, such as imatinib treatment for chronic myelogenous leukemia (CML) (75–77). In this study, we have uncovered several mutations in KDM6A, specifically H101D & P110S, N1156T and D1216N, identified in ESCC patients, increase the capability of interaction with SND1, leading to genotoxin resistance. Conversely, knockdown of both KDM6A and SND1 in ESCC cells attenuates resistance to genotoxin. In line with a previous finding that KDM6A confers imatinib-resistance in CML independently of its demethylase activity [76], our study shows that catalytic-dead KDM6A mutant promotes CPT resistance in ESCC cells and xenograft model. Thus, targeting KDM6A mutations that specifically interacting with SND1 in ESCC can be exploited as cancer cell-specific therapeutic strategies for chemotherapy.

In summary, our findings propose a model wherein KDM6A plays a crucial role in maintaining genomic stability by its interaction with SND1. This interaction promotes the recruitment of Ku70 and RPA to nascent DNA, thereby preventing nascent DNA degradation in a manner independent of its demethylase activity. Moreover, our findings demonstrate that increased interaction between KDM6A and SND1 confers chemoresistance in ESCC (Figure 9H). Thus, targeting KDM6A and/or disrupting the interaction between KDM6A and SND1 could represent a promising therapeutic strategy for cancer treatment and overcoming the chemoresistance bottleneck.

## Supplementary Material

gkae487_Supplemental_File

## Data Availability

All data needed to evaluate the conclusions in the paper are present in the paper and/or the Supplementary Materials.

## Notes

Present address: Jian Wu, Department of Thoracic Surgery, Affiliated Hospital of Southwest, Medical University, Luzhou 646000, China.
