# Correction to ‘KDM6A–SND1 interaction maintains genomic stability by protecting the nascent DNA and contributes to cancer chemoresistance’

**DOI:** 10.1093/nar/gkae581

**Published:** 2024-06-27

This is a correction to: Jian Wu, Yixin Jiang, Qin Zhang, Xiaobing Mao, Tong Wu, Mengqiu Hao, Su Zhang, Yang Meng, Xiaowen Wan, Lei Qiu, Junhong Han, KDM6A–SND1 interaction maintains genomic stability by protecting the nascent DNA and contributes to cancer chemoresistance, *Nucleic Acids Research*, 2024, gkae487, https://doi.org/10.1093/nar/gkae487

Funding from the National Key Research and Development Program of China has been corrected to 2022YFA1103702.

The published article has been updated.

