# Supplementary material for: KDM6A–SND1 interaction maintains genomic stability by protecting the nascent DNA and contributes to cancer chemoresistance
Source: Nucleic Acids Res. 2024 Jun 8;52(13):7665–86. doi: 10.1093/nar/gkae487 (PMC11260493; doi:10.1093/nar/gkae487)
Supplement: gkae487_Supplemental_File [file gkae487_supplemental_file.pdf]

Supplementary Figures and Figure Legends

Fig. S1 The colocalization of KDM6A with RPA1 in HeLa cells treated with CPT. Representative images with scale bars (5 μm) are shown.

Fig. S2 KDM6A could recruit Ku70 protein on nascent DNA through TPR domain. (A) CPT treatment could enhance the interaction of KDM6A with Ku70. Co-IP was performed in cells treated with CPT followed by western blot with indicated antibodies. (B) The association of KDM6A with Ku70 in HeLa cells treated with CPT. Representative images with scale bars (5 μm) are shown. (C) Detection of the interaction between SND1 either with Ku70 or PCNA. SND1 was immunoprecipitated using specific antibodies against SND1, followed by western blot with indicated antibodies. (D) Co-IP confirmed that TPR domain mediated the interaction of KDMA6A and Ku70. (E) SND1 knockdown attenuated the enrichment of KDM6A and Ku70 on nascent DNA which could be restored by wild-type SND1 and TSNΔ truncation.

Fig. S3. **KDM6A regulated the stability of replication forks through SUMOylation.** (A) Predicted SUMOylation sites in KDM6A protein. (B) BrdU and EdU were incorporated into nascent DNA for observing the formation of replication forks foci in HeLa cells. The histogram showed the number of replication foci formation in indicated cells. *$p < 0.05$.

Fig. S4. The characteristic of KDM6A expression in various tumors. (A) A pancancer analysis of KDM6A expression using TCGA data base. (B) KDM6A expression and mutations in ESCC was analyzed using data from public database Cbioportal. *$p < 0.05$, **$p < 0.01$. **$p < 0.001$.

Fig. S5. KDM6A mutation involved in the genomic stability of ESCC. (A) Cellular viability in the presence of genotoxin CPT for ESCC Kyse150 cells knocked down

endogenous KDM6A and followed by transfection with the plasmid of KDM6Awt or KDM6A mutants respectively. (B) Western blot for phospho-RPA32$^{S4/S8}$ and γH2AX detection in ESCC cells lacking KDM6A with KDM6Awt or KDM6A mutant plasmid transfection respectively.

Figure S1

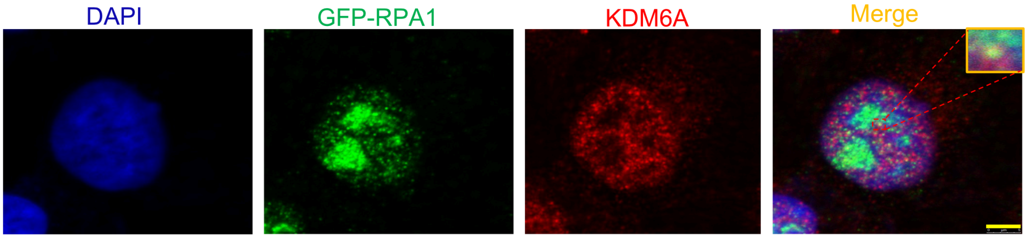

Figure S2

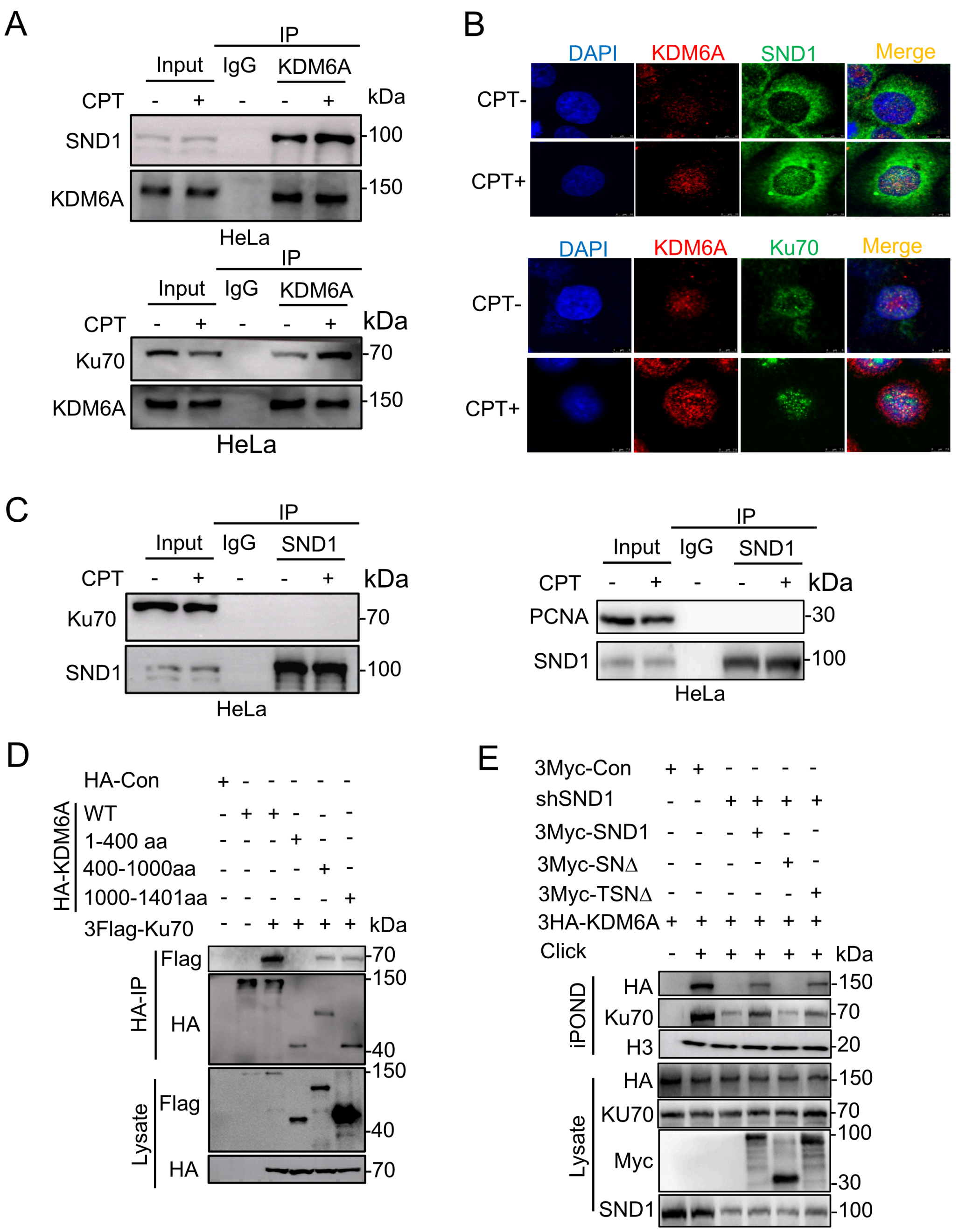
A
IP
Input
IgG
KDM6A
CPT - + - - +
kDa
SND1
KDM6A
100
150
HeLa
Ku70
70
HeLa
B
DAPI
KDM6A
SND1
Merge
CPT-
CPT+
Ku70
C
SND1
PCNA
30
D
HA-Con
HA-KDM6A
WT
1-400 aa
400-1000aa
1000-1401aa
3Flag-Ku70
HA-IP
Lysate
Flag
HA
40
E
3Myc-Con
shSND1
3Myc-SND1
3Myc-SNΔ
3Myc-TSNΔ
3HA-KDM6A
Click
iPOND
H3
20
KU70
Myc

Figure S3

A

Results for putatifs SUMO site								
Position K	Sequence	Best PS	Consensus direct			Consensus Inverted		
			Type	PSd	DB Hit	Type	PSi	DB Hit
K2	---------MKSCGVSLATAA	None	None	None		None	None	1
K23	AAAAAFGDEEKKMAAGKASGE	Low	None	None		None	Low	
K24	AAAAFGDEEKKMAAGKASGES	Low	None	None		Consensus inv	Low	
K86	AVRCYESLILKAEGKVESDFF	Low	NDSM	Low	8	None	None	
K90	YESLILKAEGKVESDFFCQLG	High	Weak Consensus	None	2	Strong consensus inv	High	
K111	HFNLLLEDYPKALSAYQRYYS	None	None	None		None	None	1
K151	FHYNAFQWAIKAFQEVLYVDP	None	None	None		None	None	1
K242	LQTENLSAQVKATVLQQLGWM	None	None	None		None	None	2
K265	TVDLLGDKATKESYAIQYLQK	None	None	None		None	None	1
K275	KESYAIQYLQKSLEADPNSGQ	None	None	None		None	None	1
K299	FLGRCYSSIGKVQDAFISYRQ	None	None	None		None	None	2
K313	AFISYRQSIDKSEASADTWCS	None	None	None	1	None	None	
K716	SGGQQGITLTKESKPSGNILT	None	None	None		None	None	1
K719	QQGITLTKESKPSGNILTVPE	High	None	None		Consensus inv	High	
K867	EGMEESQSPMKTDLLLVNHKP	Low	Consensus	Low		None	None	
K905	EVLKACRNLGKNGLSNSSILL	None	None	None		None	None	1
K978	TVIRGLAGALKLDLGLFSTKT	Low	Strong Consensus	Low		None	None	1
K1076	TSSDNSGRRRKGPFKTIKFGT	None	None	None		None	None	1
K1095	GTNIDLSDDKKWKLQLHELTK	None	None	None		Consensus inv	None	
K1265	RYEWNKLQSVKSIVPMVHLSW	None	None	None		None	None	1
K1287	MARNIKVSDPKLFEMIKYCLL	Low	None	None		Strong consensus inv	Low	
K1301	MIKYCLLRTLKQCQTLREALI	None	None	None		None	None	3
K1324	GKEIIWHGRTKEEPAHYCSIC	None	None	None	1	None	None	
K1379	LENFVVLEQYKMEDLMQVYDQ	None	Weak Consensus	None		None	None	

B

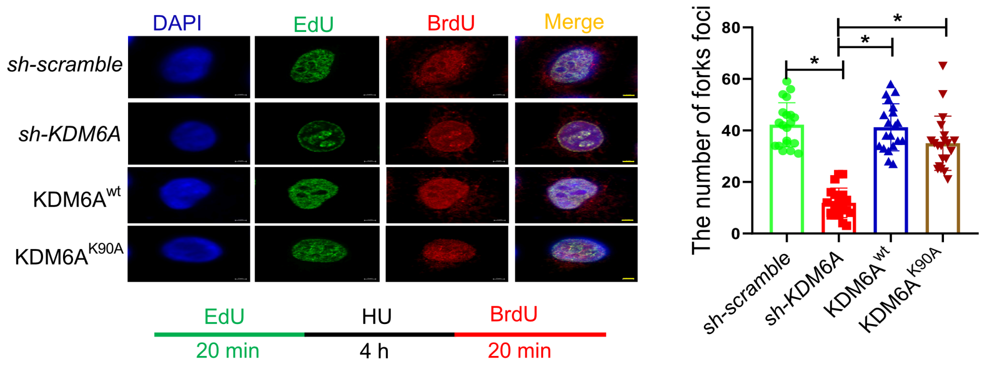

Figure S4

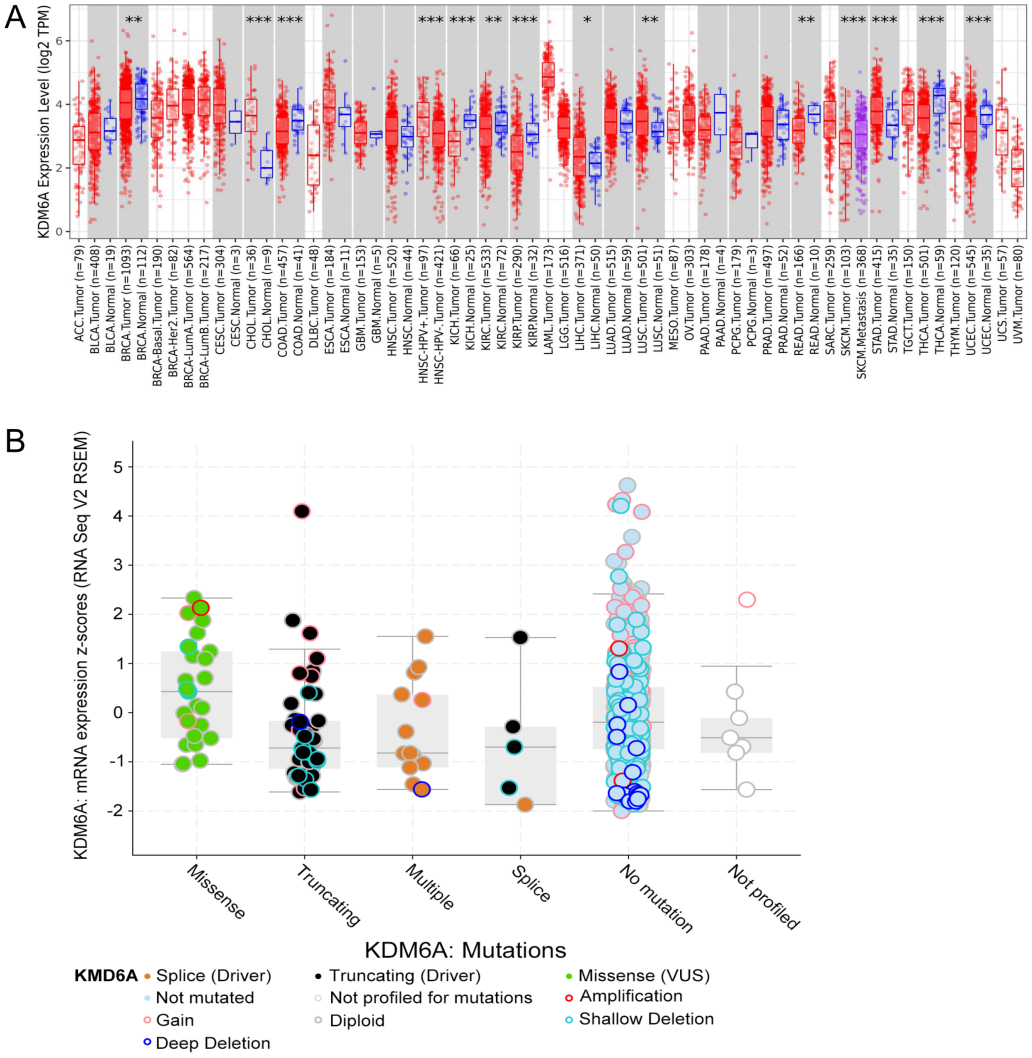
A
KDM6A Expression Level (log2 TPM)
B
KDM6A: mRNA expression z-scores (RNA Seq V2 RSEM)
Missense
Truncating
Multiple
Splice
No mutation
Not profiled
KDM6A: Mutations
KMD6A
Splice (Driver)
Truncating (Driver)
Missense (VUS)
Not mutated
Not profiled for mutations
Amplification
Gain
Diploid
Shallow Deletion
Deep Deletion

Figure S5

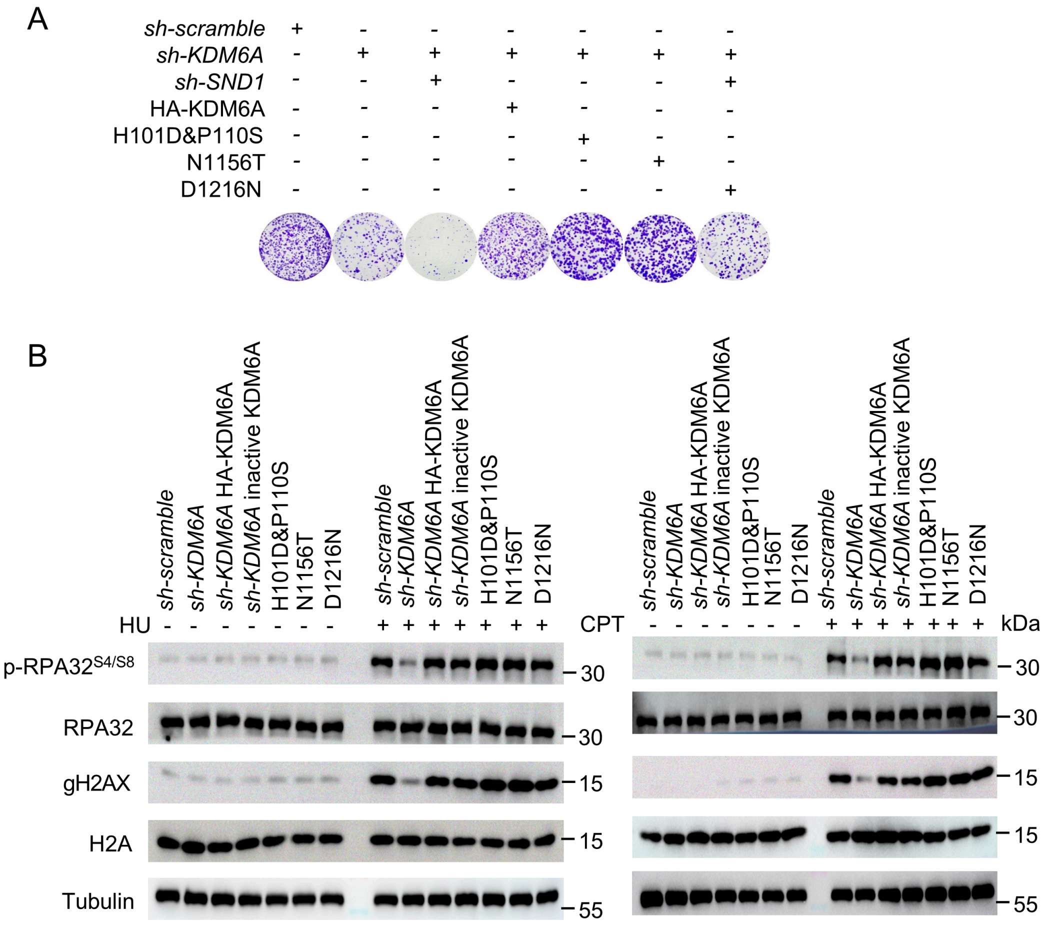
A
sh-scramble + - - - - - -
sh-KDM6A - + + + + + +
sh-SND1 - - + - - - +
HA-KDM6A - - - + - - -
H101D&P110S - - - - + - -
N1156T - - - - - + -
D1216N - - - - - - +
B
sh-scramble
sh-KDM6A
sh-KDM6A HA-KDM6A
sh-KDM6A inactive KDM6A
H101D&P110S
N1156T
D1216N
HU
CPT
kDa
p-RPA32S4/S8
RPA32
gH2AX
H2A
Tubulin
30
15
55
